# Identification of Group A Streptococcus Genes Directly Regulated by CsrRS and Novel Intermediate Regulators

**DOI:** 10.1128/mBio.01642-21

**Published:** 2021-07-13

**Authors:** Meredith B. Finn, Kathryn M. Ramsey, Simon L. Dove, Michael R. Wessels

**Affiliations:** a Division of Infectious Diseases, Boston Children’s Hospital, Boston, Massachusetts, USA; b Department of Pediatrics, Harvard Medical School, Boston, Massachusetts, USA; c Department of Cell and Molecular Biology, University of Rhode Island, Kingston, Rhode Island, USA; d Department of Biomedical and Pharmaceutical Sciences, University of Rhode Island, Kingston, Rhode Island, USA; Nanyang Technological University

**Keywords:** *Streptococcus pyogenes*, chromatin immunoprecipitation, two-component system, virulence regulation

## Abstract

Adaptation of group A Streptococcus (GAS) to its human host is mediated by two-component systems that transduce external stimuli to regulate bacterial physiology. Among such systems, CsrRS (also known as CovRS) is the most extensively characterized for its role in regulating ∼10% of the GAS genome, including several virulence genes. Here, we show that extracellular magnesium and the human antimicrobial peptide LL-37 have opposing effects on the phosphorylation of the response regulator CsrR by the receptor kinase CsrS. Genetic inactivation of CsrS phosphatase or kinase activity, respectively, had similar but more pronounced effects on CsrR phosphorylation compared to growth in magnesium or LL-37. These changes in CsrR phosphorylation were correlated with the repression or activation of CsrR-regulated genes as assessed by NanoString analysis. Chromatin immunoprecipitation and DNA sequencing (ChIP-seq) revealed CsrR occupancy at CsrRS-regulated promoters and lower-affinity associations at many other locations on the GAS chromosome. Because ChIP-seq did not detect CsrR occupancy at promoters associated with some CsrR-regulated genes, we investigated whether these genes might be controlled indirectly by intermediate regulators whose expression is modulated by CsrR. Transcriptional profiling of mutant strains deficient in the expression of either of two previously uncharacterized transcription regulators in the CsrR regulon indicated that one or both proteins participated in the regulation of 22 of the 42 CsrR-regulated promoters for which no CsrR association was detected by ChIP-seq. Taken together, these results illuminate CsrRS-mediated regulation of GAS gene expression through modulation of CsrR phosphorylation, CsrR association with regulated promoters, and the control of intermediate transcription regulators.

## INTRODUCTION

Group A Streptococcus (S. pyogenes or GAS) remains a major public health threat despite its continued susceptibility to penicillin and other beta-lactam antibiotics. GAS is responsible for millions of cases annually of pharyngitis and superficial skin infections as well as less common but life-threatening invasive infections such as septicemia, necrotizing soft tissue infection, and streptococcal toxic shock syndrome. Invasive infections and sequelae of the postinfectious syndrome of acute rheumatic fever together account for more than 500,000 deaths each year (1). Human beings are the exclusive natural host for GAS, and the persistence of GAS colonization and disease within human populations throughout the world reflects the exquisite adaptation of this pathobiont for survival in the human host. Such adaptation is facilitated by two-component regulatory systems (TCSs) that transduce signals from the environment to induce adaptive changes in bacterial physiology that enhance bacterial survival.

The CsrRS (or CovRS) TCS regulates the expression of approximately 10% of GAS genes (2–6). While the genes included in the CsrRS regulon vary somewhat in different strains, the core regulon includes genes encoding important virulence determinants such as the hyaluronic acid capsule biosynthetic enzymes, streptolysin O and its cotoxin NAD glycohydrolase (NADase), the interleukin-8 (IL-8) protease SpyCEP, streptokinase, the integrin-like IgG protease Mac/IdeS, the major secreted protease SpeB, and protein G-related α_2_-macroglobulin binding protein (GRAB). The critical role of CsrRS regulation in virulence has been highlighted by the observation that up to 40% of streptococcal toxic shock isolates harbor spontaneous mutations in *csrR* or (more commonly) *csrS*. Loss-of-function mutations in either protein are associated with the derepression of most CsrRS-regulated genes, increased virulence in mice, and increased resistance to opsonophagocytic killing in human blood (5, 7–9).

Such inactivating mutations are rarely identified among noninvasive throat isolates of GAS, which implies that a functional CsrRS system is adaptive for GAS survival in its normal habitat, the human throat (9). Prior studies from our group and others have identified two environmental stimuli that signal through the CsrRS system (3, 10–14). High concentrations of extracellular magnesium result in the repressed expression of most CsrRS-regulated genes, whereas subinhibitory concentrations of the human cathelicidin antimicrobial peptide LL-37 have the opposite effect. While magnesium concentrations in human extracellular fluids are tightly regulated at ∼1 mM, local concentrations of LL-37 vary widely. Because the secretion of LL-37 by immune and epithelial cells occurs in response to bacterial products and host cell injury, we have proposed that CsrRS-mediated upregulation of virulence gene expression in response to LL-37 represents a GAS adaptation to resist clearance by host immune effectors. In particular, exposure to physiological concentrations of LL-37 triggers increased GAS production of multiple antiphagocytic factors and results in a striking increase in GAS resistance to phagocytic killing by human neutrophils (11).

While the importance of the CsrRS system in GAS virulence is well established, less is known about the molecular mechanisms of how environmental cues signal through the system to control gene expression. How do extracellular magnesium and LL-37 modulate CsrRS-regulated gene expression? Is CsrRS regulation mediated by the physical association of CsrR with target gene promoters? To what extent is CsrRS regulation mediated by modulation of the expression of other transcription regulators? In order to answer these and related questions, we investigated the effects of extracellular signals and targeted mutations on CsrS signal transduction, CsrR phosphorylation, CsrR association with the GAS chromosome, and CsrRS-regulated gene expression. The findings reveal new insight into the molecular basis of CsrRS regulation and its intersection with other important GAS regulatory systems.

## RESULTS

### RNA-seq defines the CsrRS regulon in M1 strain 854.

While several well-known virulence factors appear to be regulated by CsrRS in most strains, the repertoire of regulated genes varies among and within GAS serotypes (2–4, 15, 16). To define the CsrRS regulon in a clinical isolate representative of the globally dominant GAS M1T1 clonal group, we performed transcriptome sequencing (RNA-seq) analysis comparing wild-type strain 854 to its *csrR* deletion mutant at mid-exponential phase. The analysis revealed a total of 118 genes with at least a 2-fold-higher or -lower transcript abundance in the mutant than in the wild-type strain (Table 1; see also Table S1 in the supplemental material). Because 47 CsrR-regulated genes were located in 15 putative operons, the total number of regulated transcription units was 87. As expected, these included multiple genes previously identified as being part of the CsrRS regulon in other GAS strains (2–6, 17). Of the 87 regulated loci, 59 exhibited increased transcript abundances in the *csrR* mutant, consistent with the generally accepted model that CsrR acts primarily as a transcription repressor, and for 28 loci, the transcript abundance was reduced in the *csrR* mutant.

**TABLE 1:** Selected virulence factors regulated by CsrR as determined by RNA-seq

Gene locus	Gene name	Gene product	Log_2_ fold change of Δ*csrR* mutant vs WT^*a*^	Adjusted *P* value
Spy_0139	*nga*	NAD glycohydrolase	3.12	3.90E−12
Spy_0141	*slo*	Streptolysin O	2.55	2.10E−10
Spy_0341	*scpC-prtS*	IL-8 protease	2.68	2.08E−15
Spy_0351	*spyA*	C3 family ADP-ribosyltransferase	3.02	9.61E−32
Spy_0356	*speJ*	Pyrogenic exotoxin type J	1.84	9.15E−04
Spy_0562	*sagA*	Streptolysin S	1.82	1.59E−09
Spy_0667	*speC*	Pyrogenic exotoxin type C	9.06	1.72E−09
Spy_0668	*mac*	IgG-degrading protease	10.01	1.45E−15
Spy_0996	*speA2*	Pyrogenic exotoxin A2	2.54	9.97E−08
Spy_1169	*spd3*	DNase	1.23	4.58E−03
Spy_1275	*arcA*	Arginine deiminase	1.12	2.42E−03
Spy_1415	*sdaD2*	Phage-encoded DNase	3.28	4.44E−15
Spy_1684	*ska*	Streptokinase	4.32	7.70E−65
Spy_1687	*sclA*	Collagen-like surface protein	7.75	9.53E−07
Spy_1715	*scpA*	C5A peptidase	1.76	6.10E−12
Spy_1718	*sic1.01*	Inhibitor of complement protein	2.99	1.13E−56
Spy_1851	*hasA*	Hyaluronan synthase	6.41	3.57E−89

a WT, wild type.

10.1128/mBio.01642-21.2 TABLE S1 CsrR regulon as determined by RNA-seq. Download Table S1, PDF file, 0.1 MB. Copyright © 2021 Finn et al. 2021 Finn et al. https://creativecommons.org/licenses/by/4.0/ This content is distributed under the terms of the Creative Commons Attribution 4.0 International license.

### Mg and LL-37 modulate CsrR phosphorylation by CsrS.

**FIG 1:**
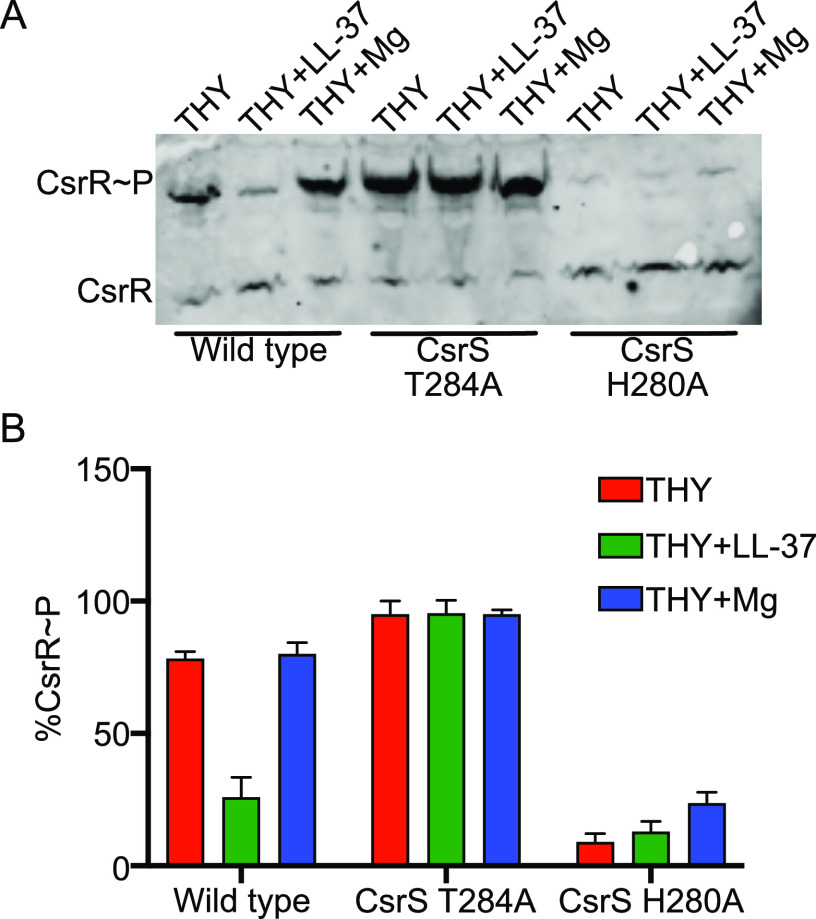
Effect of environmental signals and *csrS* mutations on CsrR phosphorylation. (A) Representative Western immunoblot for CsrR for GAS lysates separated by electrophoresis on a 10% Phos-tag polyacrylamide gel. Strains tested were wild-type GAS 854, CsrS phosphatase mutant T284A, and CsrS kinase mutant H280A, all grown in unsupplemented liquid medium (THY) or medium supplemented with 300 nM LL-37 or 15 mM MgCl_2_. CsrR∼P denotes phosphorylated CsrR protein. (B) Densitometric quantification of the percentage of total CsrR represented by CsrR∼P in the indicated strains and growth conditions. Values represent means ± standard errors of the means (SEM) of data from three independent experiments.

Growth of GAS in the presence of 10 to 20 mM magnesium has been shown to repress the expression of most CsrRS-regulated genes (3, 10). This effect is dependent on a functional CsrS and is associated with increased phosphorylation of CsrR (10, 13). Conversely, exposure of GAS to the human antimicrobial peptide LL-37 at a concentration of 100 to 300 nm has an opposite effect, increasing the transcript abundances of most CsrRS-regulated genes in a CsrS-dependent manner, and is associated with reduced phosphorylation of CsrR (11, 13). Both magnesium and LL-37 are thought to bind to the extracellular domain of CsrS and to modulate CsrR phosphorylation by stimulating or inhibiting CsrS kinase and/or phosphatase activities (3, 12). We tested this model by growing wild-type strain 854 in unsupplemented broth or in the same medium supplemented with either 15 mM MgCl_2_ or 300 nM LL-37. We then separated phosphorylated from unphosphorylated CsrR in bacterial lysates using Phos-tag gel electrophoresis and analyzed CsrR phosphorylation by Western blotting with an anti-CsrR antibody. As reported previously for a different M1 isolate, growth in the presence of supplemental magnesium was associated with only a minimal increase in the abundance of phosphorylated CsrR (CsrR∼P) relative to unphosphorylated CsrR, while the addition of LL-37 resulted in a marked decrease in CsrR∼P (Fig. 1) (13). Conversely, in a previous study, similar assays in an M3 background showed a marked increase in the abundance of CsrR∼P in the presence of supplemental magnesium and only a minor decrease in CsrR∼P in the presence of LL-37 (13). Despite the relatively small change in CsrR∼P abundance observed in strain 854 during growth in supplemental magnesium in the current study, both magnesium and LL-37 had readily demonstrable (and opposite) effects on CsrR-regulated gene expression (see below).

Consistent with the observations of Horstmann et al., we found that alanine substitution of CsrS histidine 280, the site of autophosphorylation of CsrS, almost completely prevented the phosphorylation of CsrR, regardless of the presence or absence of magnesium or LL-37 (Fig. 1) (13). Alanine substitution of threonine 284, required for the phosphatase activity of CsrS, resulted in a high level of CsrR phosphorylation, which was unaffected by magnesium or LL-37. Taken together, these data demonstrate that magnesium and LL-37 have opposing effects on the phosphorylation of CsrR by CsrS and that genetic inactivation of CsrS phosphatase or kinase activity, respectively, results in similar but more pronounced effects on the phosphorylation state of CsrR.

### The phosphorylation state of CsrR regulates the expression of the CsrRS regulon.

**FIG 2:**
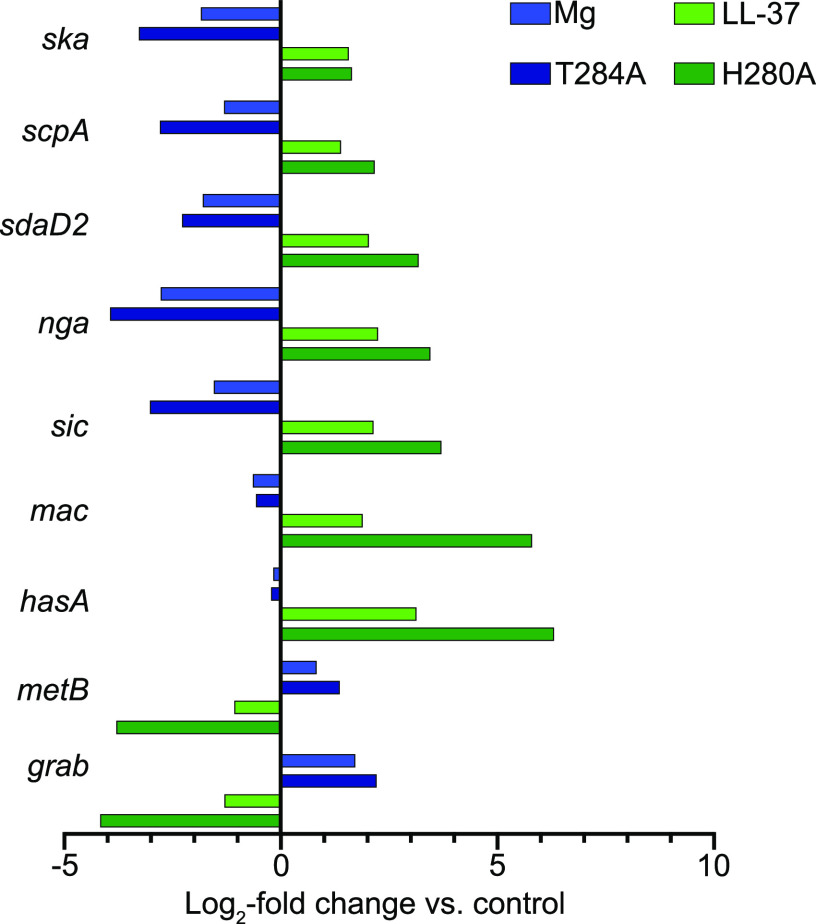
Effect of environmental signals and *csrS* mutations on the expression of CsrR-regulated genes. The transcript abundances of selected genes in the CsrR regulon were determined by NanoString analysis. RNA was isolated from GAS 854, CsrS phosphatase mutant T284A, CsrS kinase mutant H280A, or wild-type 854 grown in THY medium supplemented with 15 mM MgCl_2_ or 300 nM LL-37, all grown to mid-exponential phase (*A*_600_ of ∼0.4).

Since both exposure of GAS to magnesium and genetic inactivation of CsrS phosphatase activity result in increased phosphorylation of CsrR, we predicted that growth in magnesium or mutation of CsrS threonine 284 would have similar effects on the expression of the CsrRS regulon. Similarly, we anticipated that exposure of GAS to LL-37 would have effects on the expression of CsrRS-regulated genes similar to those of mutation of CsrS histidine 280, which is required for CsrS autokinase activity. To test this hypothesis, we performed NanoString analysis using a probe set of 179 genes that included CsrR-regulated genes identified in our RNA-seq analysis, genes associated with prominent CsrR enrichment peaks in chromatin immunoprecipitation and DNA sequencing (ChIP-seq) analysis (see below), and additional CsrR-regulated genes identified in previous microarray studies. In general, the gene expression data from the NanoString analysis showed excellent agreement with the RNA-seq data (Table S2). NanoString also identified a significant 2-fold or higher change in expression in 854ΔCsrR relative to the wild type for 12 genes not identified by RNA-seq (Table S2). We observed that the pattern of changes in the transcript abundances of target genes in the CsrS H280A mutant relative to the wild type was similar to that of the wild-type strain grown in LL-37 relative to growth in unsupplemented medium (Fig. 2; Table S3). Likewise, the pattern of gene expression changes in the CsrS T284A mutant relative to the wild type was similar to that of the wild-type strain grown in magnesium (Fig. 2; Table S4). While the patterns of regulation were similar in response to LL-37 or inactivation of CsrS kinase activity and in response to magnesium or inactivation of CsrS phosphatase activity, the magnitude of gene activation or repression was higher for the mutant strains than that in response to exposure of the wild type to LL-37 or magnesium (Fig. 2; Tables S3 and S4). This relationship mirrors the extreme effect on CsrR phosphorylation in the CsrS point mutants compared to those associated with exposure of the wild type to LL-37 or magnesium (Fig. 1). Taken together, these results support the model that environmental signals act through CsrS to decrease or increase the phosphorylation of CsrR and that the phosphorylation state of CsrR controls the repression or activation of CsrRS-regulated genes.

10.1128/mBio.01642-21.3 TABLE S2 List of genes included in NanoString studies. Download Table S2, PDF file, 0.10 MB. Copyright © 2021 Finn et al. 2021 Finn et al. https://creativecommons.org/licenses/by/4.0/ This content is distributed under the terms of the Creative Commons Attribution 4.0 International license.

10.1128/mBio.01642-21.4 TABLE S3 NanoString analysis of genes differentially transcribed in a CsrS H280A mutant or during growth of wild-type strain 854 in the presence of 300 nM LL-37. Download Table S3, PDF file, 0.07 MB. Copyright © 2021 Finn et al. 2021 Finn et al. https://creativecommons.org/licenses/by/4.0/ This content is distributed under the terms of the Creative Commons Attribution 4.0 International license.

10.1128/mBio.01642-21.5 TABLE S4 NanoString analysis of genes differentially transcribed in a CsrS T284A mutant or during growth of wild-type strain 854 in the presence of 15 mM MgCl_2_. Download Table S4, PDF file, 0.07 MB. Copyright © 2021 Finn et al. 2021 Finn et al. https://creativecommons.org/licenses/by/4.0/ This content is distributed under the terms of the Creative Commons Attribution 4.0 International license.

### CsrR phosphorylation enhances the repression of most CsrR-regulated genes.

As discussed above, LL-37 and magnesium have opposing effects on the expression of CsrR-regulated genes (Fig. 2). The expression of most CsrR-regulated genes is repressed by the exposure of GAS to elevated magnesium, and expression is activated in the presence of LL-37. Genes exhibiting this pattern of regulation have been termed group I genes and include targets such as *hasABC*, *nga*, *ska*, and *scpA* (13). This pattern of regulation is compatible with a model in which CsrR acts as a transcription repressor and its binding affinity for target promoters is increased by phosphorylation. Alternatively, or in addition, the phosphorylation of CsrR may promote the oligomerization of CsrR, thereby enhancing its activity as a transcription repressor (18). A smaller subset of genes exhibits the reverse phenotype. That is, their expression is repressed in response to LL-37 and derepressed in magnesium. Genes in the latter group, which have been called group II genes, include *metB*, *grab*, and, in strain 854, *csrR* itself (Fig. 2). At least two nonexclusive mechanisms might explain the latter pattern of regulation: (i) group II gene promoters may have a higher binding affinity for unphosphorylated CsrR, or (ii) their regulation by CsrRS may be indirect, i.e., mediated by another regulator whose expression is controlled by CsrRS. Several known or putative transcription regulators are included in the CsrRS regulon of GAS 854, and one or more of these might serve such a function.

### ChIP-seq reveals features of CsrR occupancy on the GAS chromosome.

**FIG 3:**
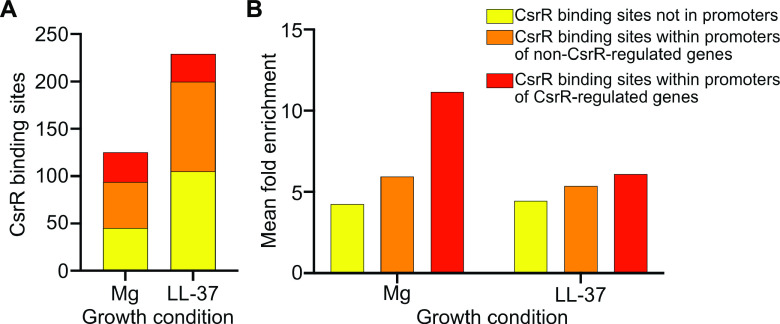
ChIP-seq analysis identifies CsrR binding sites on the GAS strain 854 chromosome. (A) Quantification and localization of CsrR binding sites in GAS strain 854 grown in medium supplemented with 15 mM MgCl_2_ or 300 nM LL-37. (B) Mean relative enrichment of DNA sequences bound by CsrR at chromosomal sites within or outside promoter regions.

**FIG 4:**
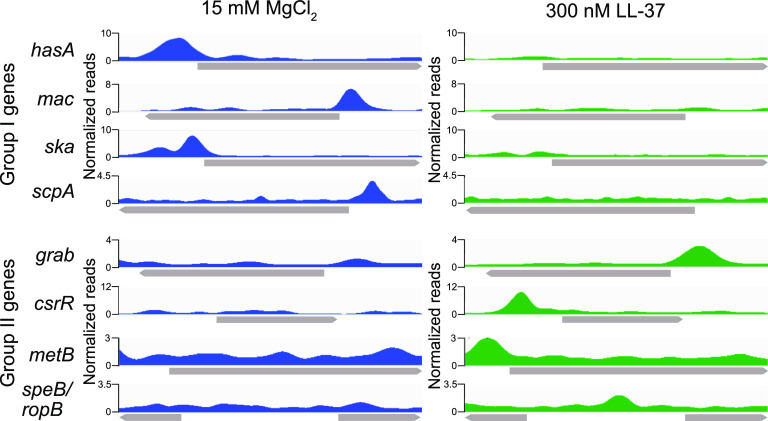
ChIP-seq analysis reveals distinct patterns of CsrR binding to promoters of regulated genes. Colored areas represent the density of normalized sequencing reads near selected genes and reflect preferential CsrR binding to promoters of group I genes during growth in supplemental magnesium and to promoters of group II genes during growth in supplemental LL-37. Genes are indicated by gray arrows. For the *speB*-*ropB* locus, the direction of *speB* transcription is to the left, and the direction of *ropB* transcription is to the right.

In order to further verify our model that CsrRS regulation is mediated by CsrR binding to target gene promoters and to resolve the mechanism(s) reflected in the regulation of group II genes, we used ChIP-seq to evaluate CsrR association with the GAS chromosome. To perform such experiments, we introduced a FLAG epitope tag as a C-terminal fusion to CsrR expressed from its native chromosomal locus in strain 854. CsrR-DNA complexes were immunoprecipitated from GAS lysates with anti-FLAG antibody and processed for DNA sequencing. After growth of strain 854*csrR-*FLAG in 15 mM magnesium, we observed CsrR occupancy at 125 chromosomal regions (Table S5). Of these, 31 (25%) were in putative promoters upstream of genes shown to be regulated by CsrR in RNA-seq analysis (Fig. 3A). Growth in 300 nM LL-37 resulted in 229 regions of CsrR occupancy, of which 28 (13%) were in CsrR-regulated promoters (Fig. 3A; Table S6). Among CsrR-regulated promoters, 18 exhibited CsrR occupancy in both magnesium and LL-37, 14 exhibited CsrR occupancy only in magnesium, and 13 exhibited CsrR occupancy only in LL-37. We also analyzed the depth of sequence reads at each CsrR binding site. This analysis revealed only a modest mean peak enrichment (i.e., read depth) for CsrR association with sites outside promoter regions, 4.5-fold and 4.2-fold for growth in LL-37 and magnesium, respectively. Somewhat greater mean peak enrichment was observed for CsrR association sites within promoters of genes not regulated by CsrR, 5.3- and 6-fold, respectively, and even greater enrichment was observed for sites within promoters of CsrR-regulated genes, 7- and 12-fold (Fig. 3B). These results suggest that although CsrR associated with many sites on the chromosome, occupancy is generally more avid at promoters of CsrR-regulated genes. Comparison of CsrR occupancy at regulated promoters during growth in magnesium versus LL-37 reveals somewhat greater average enrichment in magnesium, consistent with the model that most CsrR-regulated promoters are maximally repressed by CsrR∼P. Notably, however, greater enrichment was observed during growth in LL-37 for promoters of a few genes that are repressed in the presence of LL-37, e.g., *grab*, *metB*, and *csrR* (Fig. 4). The latter pattern is consistent with these group II promoters having a higher affinity for unphosphorylated CsrR and, hence, repressed expression in the presence of LL-37.

10.1128/mBio.01642-21.6 TABLE S5 ChIP-seq analysis of CsrR binding in wild-type GAS strain 854 grown in 15 mM MgCl_2_. Download Table S5, PDF file, 0.1 MB. Copyright © 2021 Finn et al. 2021 Finn et al. https://creativecommons.org/licenses/by/4.0/ This content is distributed under the terms of the Creative Commons Attribution 4.0 International license.

10.1128/mBio.01642-21.7 TABLE S6 ChIP-seq analysis of CsrR binding in wild-type GAS strain 854 grown in 300 nM LL-37. Download Table S6, PDF file, 0.1 MB. Copyright © 2021 Finn et al. 2021 Finn et al. https://creativecommons.org/licenses/by/4.0/ This content is distributed under the terms of the Creative Commons Attribution 4.0 International license.

A previous study proposed the consensus sequence ATTARA as a CsrR binding motif on the basis of DNase footprinting assays of CsrR binding to a 277-bp DNA fragment corresponding to the promoter and 5′-coding sequence of *hasA* (19). However, an independent investigation of the same chromosomal region as well as CsrR binding sites at other regulated promoters from M1 strain MGAS166 failed to detect a specific CsrR binding motif (15). In an attempt to identify a consensus CsrR binding site, we performed an unbiased search using MEME (part of the MEME Suite tools, version 4.12.0) (20). Specifically, we searched for a motif in the 300 bp of DNA sequence surrounding the peak maximum for 30 regions with the most CsrR association under the Mg^2+^ condition. Multiple searches were performed, varying the expected site distribution parameters. No statistically significant motifs were identified, and no motifs similar to the previously identified CsrR binding site (“ATTARA”) were identified. CentriMo (another MEME Suite tool, version 5.3.3) was used to assess if the previously identified CsrR binding site is enriched in the same sequences. No significant (*P* < 0.01) enrichment of the motif was found.

### Indirect targets of CsrR regulation are affected by intermediate regulators.

**FIG 5:**
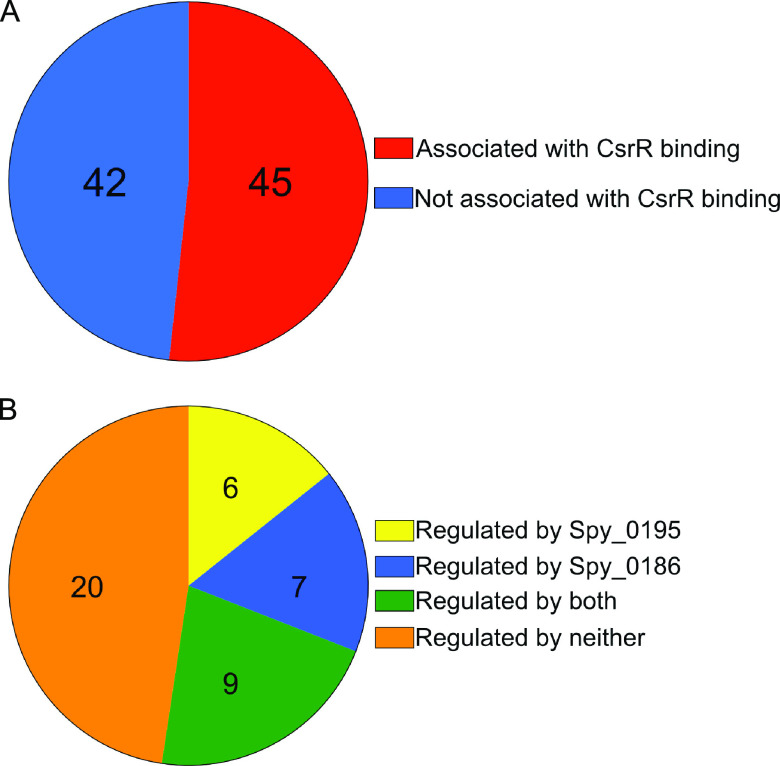
CsrR regulates gene expression directly by binding to promoters of regulated genes and indirectly by modulating the expression of other regulators. (A) Number of genes associated with CsrR binding to an upstream promoter region, as determined by ChIP-seq, among genes identified by RNA-seq analysis as belonging to the CsrR regulon. (B) Number of genes regulated by Spy_0195 or Spy_0186, as determined by NanoString analysis of mutants compared to wild-type strain 854, among genes in the CsrR regulon not associated with CsrR binding to an upstream promoter, as determined by ChIP-seq.

As discussed above, ChIP-seq analysis revealed CsrR occupancy at promoter regions of 45, or slightly more than half, of the CsrR-regulated genes identified by RNA-seq (Fig. 5A). It seems likely that many or all of the remaining CsrR-regulated genes are controlled indirectly by intermediate regulators whose expression is modulated by CsrR. Comparison of the RNA-seq and ChIP-seq data reveals at least two previously uncharacterized transcription factors that could be responsible for indirect regulation, *Spy_0186* and *Spy_0195*. Both of these genes are associated with a ChIP peak of CsrR at their promoter regions, and both exhibit altered transcript abundances in a *csrR* mutant compared to the wild type: the expression of *Spy_0195* is activated by CsrR since less transcript is present in a *csrR* mutant, while *Spy_0186* is repressed by CsrR.

To investigate further the potential role of Spy_0186 and/or Spy_0195 as an intermediate regulator of genes in the CsrRS regulon, we constructed mutants deficient in the expression of either of these two putative transcription regulators in the background of GAS strain 854. Transcription profiling of strain 854Δ0186 using the NanoString probe set showed a significant change compared to wild-type 854 in the expression of 41 of 87 genes or operons included in the CsrR regulon. Of 42 CsrR-regulated genes or operons for which no CsrR binding to an upstream promoter was detected by ChIP-seq, 16 exhibited significantly changed expression in 854Δ0186 (Fig. 5B; Table S7). We were unsuccessful in efforts to construct a deletion mutant for *Spy_0195*. Therefore, we inactivated the expression of *Spy_0195* by the insertion of the ΩKm element, which contains strong transcription terminators. We used quantitative real-time PCR (qRT-PCR) to confirm the expression of *Spy_0195* in the parent strain 854 and the absence of expression in 854-0195ΩKm. Transcription profiling experiments using strain 854-0195ΩKm showed a significant change compared to wild-type 854 in the expression of 29 genes or operons in the CsrR regulon (Fig. 5B; Table S7). Of the 42 CsrR-regulated genes or operons for which no CsrR association with an upstream promoter was detected by ChIP-seq, 15 showed significantly changed expression in 854-0195ΩKm. One or both of these regulators were implicated in the control of the expression of a number of important proteins, including xanthine phosphoribosyltransferase, an enzyme in the purine salvage pathway (21, 22); the arginine deiminase system, which mediates adaptation to acid stress and has been identified as a virulence determinant (23, 24); an uncharacterized putative transcription regulator; and several cell surface or hypothetical proteins of unknown function. These results support the hypothesis that multiple genes whose expression is indirectly modulated by CsrR are regulated through Spy_0186 or Spy_0195.

10.1128/mBio.01642-21.8 TABLE S7 Genes indirectly regulated by CsrR that are regulated by novel transcription regulators. Download Table S7, PDF file, 0.06 MB. Copyright © 2021 Finn et al. 2021 Finn et al. https://creativecommons.org/licenses/by/4.0/ This content is distributed under the terms of the Creative Commons Attribution 4.0 International license.

Both *Spy_0186* and *Spy_0195* are highly conserved among published GAS genome sequences. To assess whether the regulation of these genes by CsrR also occurs in other GAS strains, we quantified the expression of both genes by qRT-PCR in the M type 3 strain DLS003 and its *csrR* deletion mutant DLS003ΔR (7). Similar to the results observed in strain 854Δ*csrR*, the expression of *Spy_0186* was increased in DLS003ΔR compared to wild-type DLS003, and the expression of *Spy_0195* was reduced (Fig. S1). While the CsrRS regulon varies among GAS isolates, these results confirm CsrRS-mediated regulation of *Spy_0186* and *Spy_0195* in a second GAS strain.

10.1128/mBio.01642-21.1 FIG S1 Regulation of *Spy_0186* and *Spy_0195* by CsrR in GAS strains 854 and DLS003. Data represent fold changes in the transcript abundances of *Spy_0186* and *Spy_0195* as assessed by qRT-PCR in strain DLS003ΔR relative to that in DLS003 (left) or in strain 854Δ*csrR* relative to that in 854 (right). Values are means ± SEM of data from duplicate samples of three biological replicates. Download FIG S1, PDF file, 0.05 MB. Copyright © 2021 Finn et al. 2021 Finn et al. https://creativecommons.org/licenses/by/4.0/ This content is distributed under the terms of the Creative Commons Attribution 4.0 International license.

### Expression of *ropB* and *speB* is repressed by nonphosphorylated CsrR.

Another candidate intermediate regulator is RopB, also known as Rgg, which has been extensively characterized as a positive regulator of SpeB (25–28). *speB* is a group II gene; that is, CsrR represses the transcription of *speB* during growth of GAS strain 854 in the presence of LL-37 and activates its transcription during growth in supplemental magnesium (29). In our ChIP-seq analysis, we observed a moderately sized peak of CsrR association upstream of SpeB after growth in LL-37, which could be consistent with binding of unphosphorylated CsrR to the *speB* promoter and direct repression of *speB* expression (Fig. 4). The primary promoter regulating the expression of *speB* also controls the expression of a small upstream open reading frame (ORF) encoding SpeB-inducing peptide (SIP), which associates with RopB to increase the binding affinity of RopB for the *speB* promoter (27). Thus, CsrR binding to the *speB* promoter could repress *speB* expression directly and/or indirectly by repressing the expression of the SIP-encoding ORF. In addition, *ropB* is divergently transcribed from a promoter that overlaps the *speB* promoter, making it difficult to distinguish whether CsrR binding to this region directly regulates *speB* and/or *sip* or indirectly influences *speB* expression by repressing *ropB*. Our ChIP-seq results are consistent with previous reports that implicate CsrRS in the regulation of both *ropB* and *speB* and that demonstrated the binding of CsrR to a DNA probe corresponding to the *ropB*-*speB* intergenic region (18, 30, 31). By qRT-PCR analysis, we observed a significant 4-fold increase (4.0- ± 0.4-fold) in *speB* expression in 854Δ*csrR* compared to the wild type but a <2-fold increase (1.4- ± 0.04-fold) in *ropB* expression. Together, these results suggest that CsrR likely has a direct effect on *speB* and *sip* expression and may also regulate *speB* indirectly through a more modest regulatory effect on *ropB*.

### RopB affects the expression of multiple genes in the CsrRS regulon in strain 854.

**FIG 6:**
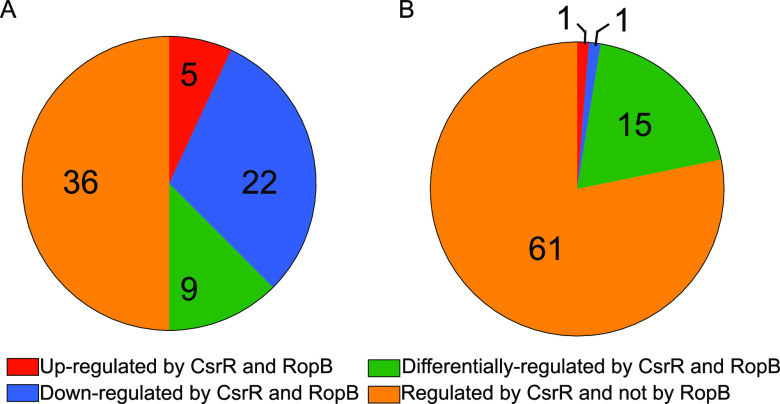
Comparison of the CsrR and RopB regulons at different growth phases. (A) Analysis of the proportion of genes within the CsrR regulon that are also influenced by RopB at mid-exponential phase. (B) Analysis of the proportion of genes within the CsrR regulon that are also influenced by RopB at stationary phase.

Previous studies have found a high degree of serotype- and strain-specific variation in the RopB regulon. While RopB regulation of *speB* and the small cotranscribed ORF encoding SIP appears to be universal, additional members of the regulon range from a single gene in M1 strain MGAS5005 to hundreds of genes in M49 strain NZ131 (32, 33). Of note, RopB was found to positively regulate *csrRS* during stationary-phase growth in strain NZ131 (32). To investigate the relationship between the CsrRS and RopB regulons in strain 854, we used our 179-gene probe set in NanoString analysis of a *csrR* mutant or a *ropB* mutant compared to the wild-type strain at mid-exponential or stationary phase. At exponential phase, a significant change in expression was observed for 67 genes in the *ropB* mutant and for 72 genes in the *csrR* mutant, 36 of which were also regulated by RopB. Of those 36 genes, 27 were regulated in similar manners in both mutants (Fig. 6A; Table S8). Thirty-one of the 36 shared genes exhibited an increase in transcript abundance in at least one of the mutant strains, and 22 of the 36 shared genes had an increase in transcript abundance in both mutants. These results are consistent with the hypothesis that both CsrR and RopB act primarily as repressors of target gene expression under these conditions. Of the 42 CsrR-regulated genes identified by RNA-seq that do not have ChIP-seq peaks, 9 were found to be regulated by RopB at mid-exponential phase. All nine of these genes also showed regulation by Spy_0186 and Spy_0195, suggesting an overlapping network of regulators responsible for the regulation of indirect targets of CsrR. We did not detect significant regulation of *csrRS* by RopB at this phase of growth.

10.1128/mBio.01642-21.9 TABLE S8 NanoString analysis of CsrR-regulated genes at mid-exponential phase and contribution of RopB to regulation. Download Table S8, PDF file, 0.07 MB. Copyright © 2021 Finn et al. 2021 Finn et al. https://creativecommons.org/licenses/by/4.0/ This content is distributed under the terms of the Creative Commons Attribution 4.0 International license.

At stationary phase, 37 genes had significant differences in transcript abundances in the *ropB* mutant compared to the wild type. In the *csrR* mutant, 79 genes had significantly different transcript abundances compared to the wild type. Of the genes with altered expression in the *ropB* mutant, 17 also had significantly changed transcript abundances in the *csrR* mutant, but only 2 were regulated in the same manner (Fig. 6B; Table S9). In general, genes that were regulated by RopB at stationary phase were activated by RopB, whereas most genes regulated by CsrR were repressed. Together, these data implicate RopB as a potential mediator of secondary regulation of multiple genes in the CsrRS regulon.

10.1128/mBio.01642-21.10 TABLE S9 NanoString analysis of CsrR-regulated genes at stationary phase and contribution of RopB to regulation. Download Table S9, PDF file, 0.07 MB. Copyright © 2021 Finn et al. 2021 Finn et al. https://creativecommons.org/licenses/by/4.0/ This content is distributed under the terms of the Creative Commons Attribution 4.0 International license.

## DISCUSSION

TCSs represent a widespread mechanism by which bacteria sense environmental conditions and respond by altering their physiology, most commonly through regulation of gene expression. While many variations exist, TCSs typically consist of a sensor kinase associated with the cell membrane and a cognate response regulator located in the bacterial cytoplasm. Interaction of extracellular stimuli with an exposed region of the sensor kinase induces a conformational change that promotes (or inhibits) the autophosphorylation of a conserved histidine in the cytoplasmic domain of the sensor protein. Interaction of the phosphorylated sensor kinase with the response regulator results in the transfer of the phosphate group to a conserved aspartic acid residue in the receiver domain of the regulator. Alternatively, or in addition, in some systems, signaling through the sensor kinase may alter its phosphatase activity for the regulator. The phosphorylation state of the regulator determines its binding affinity for promoter sequences of regulated genes and the consequent activation or repression of target gene transcription.

Several studies have provided evidence consistent with such a model for the CsrRS system in GAS. A high concentration (>10 mM) of extracellular magnesium was the first specific stimulus shown to result in CsrS-dependent repression of many CsrRS-regulated genes and was subsequently found to be associated with increased CsrR phosphorylation (3, 10, 13). We confirm those previous observations here and also show that a subinhibitory concentration of the human cathelicidin LL-37 has the opposite effect on both CsrR phosphorylation and target gene regulation, as suggested by previous studies (29, 34). We also found that the introduction of point mutations predicted to abrogate CsrS phosphatase or kinase activities resulted in similar but more extreme effects on CsrR phosphorylation and CsrRS-regulated gene expression compared to exposure of the wild-type strain to supplemental magnesium or LL-37, respectively. Together, these findings support the model that magnesium and LL-37 signal through CsrS to control its autokinase and/or phosphatase activities, thereby increasing or decreasing the phosphorylation of CsrR and, consequently, its activity as a transcription regulator.

We used ChIP-seq to investigate whether changes in gene expression triggered by signaling through CsrS were a direct result of CsrR binding at cognate promoters. These experiments revealed that CsrR (and/or CsrR∼P) associated with more than 100 sites on the GAS 854 chromosome, of which ∼25% overlapped predicted promoters of CsrRS-regulated genes. While CsrR was also found at many sites not associated with a change in gene expression, there appeared to be a hierarchy in the overall apparent avidity of CsrR association as reflected in the abundance of sequence reads: the highest avidity was for CsrR-regulated promoters, an intermediate level was observed for promoters not regulated by CsrR, and the lowest avidity of CsrR occupancy was for chromosomal sites remote from predicted promoters. The extent of association with CsrR-regulated promoters was also greater during growth in medium supplemented with magnesium than during growth in medium containing LL-37, presumably reflecting the increased affinity of CsrR∼P compared to unphosphorylated CsrR for most regulated promoters. These patterns suggest that more avid binding of CsrR is more likely to suppress gene transcription, whereas lower-avidity binding to many chromosomal sites has no obvious effect on gene expression. A similar pattern of transcription factor binding has been observed for the orphan response regulator PmrA in Francisella tularensis. By ChIP-seq analysis, PmrA associated with 252 sites on the F. tularensis chromosome, but only 18 (7%) were located in predicted promoters of genes whose expression was altered in a *pmrA* deletion mutant. Furthermore, the site with the highest degree of enrichment for PmrA corresponded to the predicted promoter of *priM*, the gene most highly regulated by PmrA (35). It has been suggested that the widespread binding of bacterial transcription factors to sites not obviously linked to transcription regulation may reflect the evolution of these proteins to a site-specific function in transcription regulation from their ancestral function as nucleoid-associated proteins, which act to shape the chromosome (36).

Most CsrRS-regulated genes, including those encoding virulence factors, are repressed during GAS growth in high magnesium and derepressed during exposure to LL-37. As expected, we observed greater binding of CsrR to such promoters during growth in magnesium, reflecting their higher relative affinity for CsrR∼P. However, previous studies and the current work have identified a few genes, termed group II, that exhibit the opposite pattern of regulation and a correspondingly higher degree of CsrR binding in the presence of LL-37 (13, 29). This result implies that promoters of the latter genes bind more avidly to unphosphorylated CsrR than to CsrR∼P. Notable among this small group of group II genes is *speB*, the expression of which is repressed by LL-37. We hypothesize that increased production of LL-37 in the human oropharynx reflects the activation of host innate immunity during GAS infection and represents a signal detected by the CsrRS system. LL-37 signaling through CsrS results in reduced phosphorylation of CsrR to derepress the expression of virulence factors that enhance GAS resistance to opsonophagocytic killing, such as the hyaluronic acid capsule, streptolysin O, NADase, the IL-8 protease SpyCEP, and the IgG protease Mac/IdeS. The secreted cysteine protease SpeB cleaves from the bacterial surface and/or degrades many secreted GAS proteins, including the critical antiphagocytic M protein as well as other virulence factors (37–42). It is teleologically appealing, therefore, to suppose that, in contrast to promoter sequences for most virulence genes, the *speB* promoter evolved to preferentially recognize unphosphorylated CsrR, ensuring the repression of protease production when antiphagocytic factors are needed to counteract the host immune response.

In addition to demonstrating the physical association of CsrR with CsrRS-regulated promoters, the current study implicated RopB as a transcriptional regulator, the expression of which is influenced by CsrR. The partial overlap of the CsrR and RopB regulons suggested that RopB may act as an intermediate regulator downstream of CsrRS, extending the CsrRS regulon to multiple genes that are transcriptionally regulated by RopB. Previous studies have found the RopB regulon to be highly variable, ranging from only its best-known target, *speB*, and a small upstream ORF to more than 100 genes (27, 31–33).

The current study also identified Spy_0186 and Spy_0195 as two previously uncharacterized putative transcription regulators whose expression is regulated by CsrR, each of which is associated with CsrR binding to an upstream promoter in ChIP-seq analysis. NanoString transcription analysis of a *Spy_0186* deletion mutant revealed significantly changed expression compared to the wild type for 16 of the 42 CsrR-regulated genes that were not associated with CsrR binding to an upstream promoter. Similarly, transcriptional profiling of a strain defective for the expression of *Spy_0195* demonstrated changed expression compared to the wild type for 15 CsrR-regulated genes not associated with CsrR binding to an upstream promoter. These results identify Spy_0186 and Spy_0195 as intermediate transcription regulators that extend the CsrRS regulon to additional target genes beyond those directly controlled by CsrR.

Inactivation of *ropB*, *Spy_0186*, or *Spy_0195* was associated with both activation and repression of a variety of genes in the CsrRS regulon, so it is unclear whether these transcription regulators can act as both direct activators and repressors or whether more complex regulatory mechanisms are involved. We did not observe convincing evidence of reciprocal regulation of CsrRS by RopB, Spy_0186, or Spy_0195, results consistent with CsrRS acting as a master regulator at the top of a regulatory cascade. Because CsrRS is a TCS that responds to environmental cues, such an arrangement is predicted to link CsrS signal transduction to a wide array of downstream targets through the direct action of CsrR and by the control of these and possibly other intermediate regulators.

In summary, our results provide new evidence that specific extracellular stimuli signal through CsrS to control the phosphorylation of CsrR. The phosphorylation state of CsrR modulates its binding affinity for multiple sites on the GAS chromosome, with the most avid binding to promoters of CsrRS-regulated genes. CsrR acts predominantly as a repressor of target gene transcription, and, for most regulated promoters, CsrR∼P appears to bind more avidly than unphosphorylated CsrR. Exceptions to this pattern may occur for promoters that bind more avidly to unphosphorylated CsrR or as a result of indirect regulation through intermediate regulators. Through these varied mechanisms, the CsrRS system senses environmental cues and transduces these signals to trigger a cascade of transcriptional responses that optimize GAS adaptation for survival in the human host.

## MATERIALS AND METHODS

### Bacterial strains and growth conditions.

Bacterial strains used in this study are listed in Table 2. The GAS strain used for molecular manipulation was 854, an M1 strain isolated from a patient with a retroperitoneal abscess (34). Gene sequences and expression analyses in strain 854 were referenced to the genome sequence of strain MGAS5005 (NCBI taxonomy identifier [txid] 293653). In order to increase the transformation efficiency of strain 854, we previously introduced a deletion mutation in *hsdM*, which encodes a component of a type I restriction-modification system. Analysis of global gene expression revealed no significant changes in the *hsdM* mutant strain 854Δ*hsdM* compared to wild-type strain 854 (43). Accordingly, strain 854Δ*hsdM* was used as the parent strain for the construction of mutant strains 854CsrR-FLAG, 854Δ*ropB*, 854Δ0186, and 854-0195ΩKm as described below. GAS strains were cultured on Trypticase soy agar supplemented with 5% defibrinated sheep blood (Remel) or Todd-Hewitt (TH) agar or in TH yeast (THY) broth at 37°C in the presence of 5% CO_2_. Where indicated, MgCl_2_ was supplemented to a final concentration of 15 mM, or LL-37 was supplemented to a final concentration of 300 nM.

**TABLE 2:** GAS strains used in this study

S. pyogenes strain	Description	Reference
854	Wild-type M1	11
854Δ*hsdM*	Deletion of *hsdM*	43
854Δ*csrR*	Deletion of *csrR*	29
854CsrS H280A	H280A mutation in CsrS	29
854CsrS T284A	T284A mutation in CsrS	50
854Δ*ropB*	Deletion of *ropB*	This study
854Δ0186	Deletion of Spy_0186	This study
854-0195ΩKm	Disruption of Spy_0195 with the ΩKm cassette	This study
854CsrR-FLAG	CsrR with a C-terminal FLAG tag	This study
DLS003	Wild-type M3	7
DLS003ΔR	Deletion of *csrR*	7

### Construction of allelic exchange vectors and GAS mutagenesis.

Oligonucleotide primers are listed in Table 3.

**TABLE 3:** Oligonucleotide primers used in this study^*a*^

Primer name	Description	Sequence^*b*^	Application
MAB154	*ropB* 5′ F-BamHI	5′-CCCC-GGATCC-GTTAATCTACTGCATTTGCTTTTA	*ropB* deletion construct
MAB155	*ropB* 5′ R	5′-ATCGTTTTGC-GCCTAGTGAGATAATCACCAC	*ropB* deletion construct
MAB156	*ropB* 3′ F	5′-CACTAGGC-GCAAAACGATCCATCATATGTT	*ropB* deletion construct
MAB157	*ropB* 3′ R-SalI	5′-CCCC-GTCGAC-ACTACCATAACGGTACACCTAC	*ropB* deletion construct
MAB173	0186 5′ F-BamHI	5′-CCCC-GGATCC-GGTCTGAATTAACAAGACTAAT	Spy_0186 deletion construct
MAB174	0186 5′ R	5′-GTTCTGGTGC-CTGGTCAATAATATCGGATTCC	Spy_0186 deletion construct
MAB175	0186 3′ F	5′-TATTATTGACCAG-GCACCAGAACCACTACCAAAA	Spy_0186 deletion construct
MAB176	0186 3′ R-SalI	5′-CCCC-GTCGAC-GTTATTCGTGTTGATAATCCTCG	Spy_0186 deletion construct
MAB248	0195 insert 5′ F Sal	5′-CCCC-GTCGAC-ATGTCACAAGTGATAGGTGATTT	Spy_0195ΩKm construct
MAB249	0195 insert 5′ R BglII	5′-CCCC-AGATCT-CAGCAACTCTTCTTTTGTAATTTC	Spy_0195ΩKm construct
MAB250	0195 insert 3′ F BglII	5′-CCCC-AGATCT-ACAGCGAAAAAAGTAATTGAACAG	Spy_0195ΩKm construct
MAB251	0195 insert 3′ R XbaI	5′-CCCC-TCTAGA-TATTGGCATATGAAAAGTCTTGG	Spy_0195ΩKm construct
MAB3	*csrR* 5′ F BamHI	5′-CCCC-GGATCC-CTACCAGTGTCTAAAAGAAGAAA	*csrR*-FLAG construct
MAB72	*csrR*-3×FLAG 5′ R	5′-TTATTTATCATGATCTTTATAATCAATATCATGATCTTTATAATCGCCATCATGATCTTTATAATCTTTCTCACGAATAACGTATCCC	*csrR*-FLAG construct
MAB80	*csrR*-3×FLAG 3′ F	5′-GATTATAAAGATCATGATGGCGATTATAAAGATCATGATATTGATTATAAAGATCATGATAAATAAGTCATATGGAAAATCAGAAACAAA	*csrR*-FLAG construct
MAB28	*csrS* 3′ R SalI	5′-CCCC-GTCGAC-TTACTAACTCTCTTTAGACTGGGC	*csrR*-FLAG construct
MAB38	qRT-PCR *speB*-F	5′-TGCAGGTAGCTCTCGTGTTC	qRT-PCR
MAB39	qRT-PCR *speB*-R	5′-GCTTCCCAATCTTGTTTGCT	qRT-PCR
MAB126	qRT-PCR *ropB*-F	5′-TTTGCCTTGGTCAAGGTGTT	qRT-PCR
MAB127	qRT-PCR *ropB*-R	5′-AGCACAGTCTCATAGTGACTCCA	qRT-PCR
MAB218	0186-qRT-PCR-F	5′-ACCTCGAACTCGTGTCACAT	qRT-PCR
MAB219	0186-qRT-PCR-R	5′-AGTGCCTGAAGACGATGGAT	qRT-PCR
MAB220	0195-qRT-PCR-F	5′-TGATGCCTTCCCAAGTGGAT	qRT-PCR
MAB221	0195-qRT-PCR-R	5′-TCCCGCAACAAAGGCAATTT	qRT-PCR
MW1	recA-qRT-PCR-F	5′-TGATTCTGGTGCGGTTTGATC	qRT-PCR
MW2	recA-qRT-PCR-R	5′-ATTTACGCATGGCCTGACTC	qRT-PCR

a F, forward; R, reverse.

b Hyphens flank non-contiguous sequence used for overlap PCR or non-coding sequence added to introduce a restriction endonuclease site.

### (i) 854CsrR-FLAG.

Using strain 854 genomic DNA as the template, we used primers MAB3 and MAB72 to PCR amplify *csrR* with the 3×FLAG sequence at the 3′ terminus. In a second reaction, primers MAB80 and MAB28 were used to amplify a portion of the *csrS* sequence with the 3×FLAG sequence at the 5′ terminus. In a third reaction, the products of reactions 1 and 2 were used as the template with primers MAB3 and MAB28 to amplify the desired fragment containing the 3×FLAG sequence flanked by *csrR* and a portion of *csrS*. This fragment was ligated into the temperature-sensitive shuttle vector pJRS233, and the recombinant plasmid was introduced into GAS strain 854 by electroporation (33). Allelic exchange with the corresponding chromosomal locus was accomplished as described previously (34).

### (ii) 854Δ*ropB*.

Using strain 854 genomic DNA as the template, we used primers MAB154 and MAB155 to PCR amplify the region immediately upstream of *ropB*. In a second reaction, primers MAB156 and MAB157 were used to amplify the region immediately downstream of *ropB*. In a third reaction, the products of reactions 1 and 2 were used as the template with primers MAB154 and MAB157 to amplify the desired fragment containing the *ropB* deletion flanked by upstream and downstream sequences. This fragment was cloned into pJRS233 for allelic exchange in strain 854 as described above for 854CsrR-FLAG.

### (iii) 854Δ0186.

Using strain 854 genomic DNA as the template, we used primers MAB173 and MAB174 to PCR amplify the region immediately upstream of *Spy_0186*. In a second reaction, primers MAB175 and MAB176 were used to amplify the region immediately downstream of *Spy_0186*. In a third reaction, the products of reactions 1 and 2 were used as the template with primers MAB173 and MAB176 to amplify the desired fragment containing the *Spy_0186* deletion flanked by upstream and downstream sequences. This fragment was cloned into pJRS233 for allelic exchange in strain 854 as described above for 854CsrR-FLAG.

### (iv) 854-0195ΩKm.

Using strain 854 genomic DNA as the template, we used primers MAB248 and MAB249 to PCR amplify the 5′ portion of *Spy_0195* and the upstream flanking sequence. In a second reaction, primers MAB250 and MAB251 were used to amplify the adjacent 3′ portion of *Spy_0195* and the downstream flanking sequence. The ΩKm cassette was obtained by digesting the vector pBR322ΩKm with the restriction enzyme BamHI. These three fragments were cloned into pJRS233 for allelic exchange in strain 854 as described above for 854CsrR-FLAG, resulting in a strain with the ΩKm cassette inserted into *Spy_0195* at nucleotide 357.

Genotypes of the mutated strains were confirmed by PCR amplification and DNA sequencing of the locus and verified by qRT-PCR for the anticipated loss of gene expression.

### Detection of CsrR phosphorylation *in vivo*.

GAS strains were grown in THY medium with either no supplement, 15 mM MgCl_2_, or 300 nM LL-37 to mid-exponential phase (optical density [OD] of ∼0.4). Cells were collected by centrifugation, resuspended in phosphate-buffered saline (PBS), and lysed by shaking with glass beads for 30 s on an amalgamator (Patterson Dental). Lysates were fractionated by electrophoresis on a 10% polyacrylamide gel containing sodium dodecyl sulfate, 100 μM Phos-tag solution (Wako Pure Chemical Industries Ltd., Richmond, VA), and 200 μM MnCl_2_ for 120 min at 180 V at 4°C. The gel was washed in transfer buffer supplemented with 1 mM EDTA for 10 min and then washed again in transfer buffer for 10 min. Proteins were then transferred to a nitrocellulose membrane, and CsrR species were detected by standard Western blotting using anti-CsrR antiserum as described previously (29).

### ChIP-seq.

ChIP-seq was performed with cells of GAS strain 854CsrR-FLAG and wild-type 854 (as a mock control). Cells were grown at 37°C to mid-exponential phase (*A*_600_ of ∼0.4) in 160 ml of THY broth either unsupplemented or supplemented with 15 mM MgCl_2_ or 300 nM LL-37. Cells were incubated in a final concentration of 1% formaldehyde (Sigma) for 30 min, after which glycine was added to a final concentration of 250 mM. A water bath sonicator (Bioruptor; Diagenode) was used to lyse cells and shear chromosomal DNA to 200 to 400 bp. ChIP was performed in biological triplicate using anti-FLAG magnetic beads (Sigma). Samples were incubated at 65°C to reverse the formaldehyde cross-links, and DNA was purified using a commercial PCR purification kit (Qiagen). Immunoprecipitated DNA was quantified using the Quanti-iT PicoGreen dsDNA (double-stranded DNA) assay kit (Invitrogen).

### ChIP-seq library preparation and sequencing.

Sequencing libraries were constructed with the NEBNext Ultra II DNA library prep kit for Illumina (New England BioLabs [NEB]) according to the manufacturer’s instructions. Approximately 25 ng of immunoprecipitated DNA was used, adapters were diluted 1:10 prior to ligation, and samples underwent 5 rounds of amplification without size selection. Libraries were sequenced by Elim Biopharmaceuticals, Inc. (Hayward, CA), on an Illumina HiSeq2500 platform, producing 50-bp paired-end reads.

### ChIP-seq data analysis.

Paired-end sequencing reads were mapped to the S. pyogenes MGAS5005 genome (NCBI RefSeq accession number NC_007297) using bowtie2 version 2.3.1 (44). Only reads corresponding to fragments of 200 bp or less were used in subsequent analyses. A custom script was used to extract only read 1 from each pair, and regions of enrichment were identified using QuEST version 2.4 (45). The appropriate mock biological replicates were merged and used as a background control for each biological replicate. The following criteria were used to identify regions of enrichment (peaks): they are enriched 2-fold or more in reads compared to the background, have a positive peak shift and strand correlation, and have a *q* value of less than 0.01. Peaks for each immunoprecipitated protein were defined as the maximal region identified in at least two biological replicates. Data were visualized using Integrative Genomics Viewer (IGV) version 2.3.9 (46). Peak analyses used custom scripts and BEDtools version 2.26.0 (47).

### RNA extraction for RNA-seq.

GAS cells were grown in THY broth to mid-exponential phase (*A*_600_ of 0.4) and collected by centrifugation. Cell pellets were resuspended in 0.5 ml TRIzol reagent (Thermo Fisher Scientific), transferred to 2-ml FastPrep tubes (MP Biomedicals) containing 0.1-mm zirconia-silica beads (BioSpec Products), and shaken for 90 s at a speed of 10 m/s using the FastPrep-24 5G system (MP Biomedicals). After the addition of 200 μl chloroform, each sample tube was mixed thoroughly by inversion, incubated for 3 min at room temperature, and subjected to centrifugation for 15 min at 4°C. The aqueous phase was mixed with an equal volume of 100% ethanol and transferred to a Direct-zol spin plate (Zymo Research), and RNA was extracted according to the Direct-zol protocol (Zymo Research).

### Generation of RNA-seq data.

Illumina cDNA libraries were generated using a modified version of the RNAtag-seq protocol (48). Briefly, 0.5 to 1 μg of total RNA was fragmented, depleted of genomic DNA, dephosphorylated, and ligated to DNA adapters carrying 5′-AN_8_-3′ barcodes of known sequence with a 5′ phosphate and a 3′ blocking group. Barcoded RNAs were pooled and depleted of rRNA using the RiboZero rRNA depletion kit (Epicentre). Pools of barcoded RNAs were converted to Illumina cDNA libraries in 2 main steps: (i) reverse transcription of the RNA using a primer designed for the constant region of the barcoded adapter with the addition of an adapter to the 3′ end of the cDNA by template switching using SMARTScribe (Clontech) as described previously (49), and (ii) PCR amplification using primers whose 5′ ends target the constant regions of the 3′ or 5′ adapters and whose 3′ ends contain the full Illumina P5 or P7 sequences. cDNA libraries were sequenced on the Illumina (NextSeq 2500) platform to generate paired-end reads.

### RNA-seq data analysis.

Paired-end sequencing reads were mapped to the S. pyogenes MGAS5005 genome (NCBI RefSeq accession number NC_007297) using bowtie2 version 2.3.1 (44). Reads that mapped to annotated genes were counted using HTSeq version 0.10.0, and analysis of differential gene expression was conducted using DESeq2 version 1.20.0. Reported genes had a 2-fold-higher or -lower abundance than the wild type, all with an adjusted *P* value of 0.05 or lower.

### NanoString nCounter assay.

Gene expression differences between wild-type 854 and 854 knockout strains or for strain 854 under different growth conditions were determined by NanoString nCounter technology (NanoString Technologies) using a custom nCounter probe library targeted to 182 different GAS genes. According to the manufacturer’s protocol, samples were prepared for hybridization with PrepStation processing, nCounter analysis system counting, and nSolver software v3.0 analysis. Transcript copies were normalized using the geometric means of 3 housekeeping genes for reference (*recA*, *proS*, and *gyrB*). The log_2_ fold change in the expression of each gene was calculated for knockout samples compared to wild-type samples or for samples of the wild type under different growth conditions.

### RNA isolation and qRT-PCR.

GAS cells were grown in THY broth, and cells were harvested at the mid-exponential (*A*_600_ of 0.4) growth phase. Total RNA extraction from bacterial cells was performed using an RNeasy minikit (Qiagen) according to the manufacturer’s instructions. RNA concentration and purity were determined using a NanoDrop ND-1000 spectrophotometer (Thermo Fisher Scientific). cDNA was generated using the high-capacity RNA-to-cDNA kit (Applied Biosystems) according to the manufacturer’s instructions.

Quantitative RT-PCR was performed on a QuantStudio 3 system (Applied Biosystems) using the PowerUp SYBR green master mix (Applied Biosystems). Primers are listed in Table 3. Triplicate assays were performed for each gene tested with 1 ng total template. Expression levels of target genes were normalized to *recA* (M5005_Spy_1799) and then further normalized to the wild-type strain expression levels to obtain a final ΔΔ*C_T_* value. These values were used to calculate relative expression changes for the mutant strain compared to the parental wild-type strain.

### Data availability.

The RNA-seq and ChIP-seq data referenced in this study are available in the National Center for Biotechnology Information Gene Expression Omnibus (GEO) under accession numbers GSE173362 and GSE173368, respectively.
