# Supplementary material for: Identification of Group A Streptococcus Genes Directly Regulated by CsrRS and Novel Intermediate Regulators
Source: mBio. 2021 Jul 13;12(4):e01642-21. doi: 10.1128/mBio.01642-21 (PMC8406183; doi:10.1128/mBio.01642-21)
Supplement: TABLE S1 [file mbio.01642-21-st001.pdf]

Table S1. CsrR regulon of GAS strain 854 as determined by RNAseq				
**Gene Locus***	**Gene name**	**Gene product**	**Log2-fold change Δ*csrR* vs. WT**	**Adjusted *p*-value**
Spy_0017	*sibA*	secreted protein	-1.04	2.57E-04
Spy_0029	*purD*	phosphoribosylamine-glycine ligase	-1.25	3.05E-02
Spy_0034	-	transcription regulator	1.37	2.75E-04
Spy_0113	-	transposase	7.17	1.30E-14
Spy_0115	-	hypothetical protein	9.45	1.94E-121
Spy_0117	-	LysR family transcription regulator	1.22	8.25E-03
Spy_0118	-	LysR family transcription regulator	1.00	3.56E-02
Spy_0129	*ntpC*	V-type ATP synthase subunit C	1.08	7.00E-02
Spy_0139	*nga*	NAD glycohydrolase	3.12	3.90E-12
Spy_0140	*ifs*	NADase inhibitor	3.00	2.42E-10
Spy_0141	*slo*	streptolysin O	2.55	2.10E-10
Spy_0143	-	hypothetical protein	11.99	7.94E-21
Spy_0144	-	hypothetical protein	9.87	8.19E-15
Spy_0155	-	BglG family transcription antiterminator	-1.03	6.73E-03
Spy_0157	*opuAA*	glycine betaine transport ATP-binding protein	-1.89	3.36E-06
Spy_0158	*opuABC*	glycine betaine-binding protein/glycine betaine transporter permease	-1.53	2.77E-05
Spy_0186	-	transcription regulator	1.67	3.07E-06
Spy_0195	-	MarR family transcription regulator	-1.69	5.54E-04
Spy_0196	-	multidrug resistance ABC transporter ATP-binding protein/permease	-1.37	2.41E-04
Spy_0197	-	multidrug resistance ABC transporter ATP-binding protein/permease	-1.09	1.45E-03
Spy_0275	-	serine/threonine transporter SstT	-2.05	1.70E-09
Spy_0282	*csrR*	response regulator	-12.95	1.78E-20
Spy_0341	*scpC/prtS*	IL-8 protease	2.68	2.08E-15
Spy_0351	*spyA*	C3 family ADP-ribosyltransferase	3.02	9.61E-32
Spy_0352	-	hypothetical protein	6.33	1.31E-37
Spy_0354	-	hypothetical protein	3.14	2.49E-22
Spy_0355	-	hypothetical protein	4.18	2.20E-02
Spy_0356	*speJ*	pyrogenic exotoxin type J	1.84	9.15E-04
Spy_0357	-	hypothetical protein	2.42	3.59E-12
Spy_0374	*rplK*	50S ribosomal protein L11	-1.04	3.98E-03
Spy_0375	*rplA*	50S ribosomal protein L1	-1.04	2.10E-04
Spy_0551	*rplS*	50S ribosomal protein L19	-1.77	4.57E-13
Spy_0557	-	transposase	1.89	4.62E-07
Spy_0558	-	transposase	1.53	1.35E-02

Spy_0559	-	transcription regulator	1.92	1.78E-05
Spy_0560		transcription regulator	1.60	1.07E-05
Spy_0561	*epf*	extracellular matrix binding protein	4.30	4.57E-45
Spy_0562	*sagA*	streptolysin S	1.82	1.59E-09
Spy_0563	*sagB*	streptolysin S biosynthesis protein	1.83	7.15E-05
Spy_0564	*sagC*	streptolysin S biosynthesis protein	2.17	6.04E-07
Spy_0565	*sagD*	streptolysin S biosynthesis protein	2.05	4.28E-07
Spy_0566	*sagE*	streptolysin S self-immunity protein	1.95	3.81E-07
Spy_0567	*sagF*	streptolysin S biosynthesis protein	2.11	3.82E-08
Spy_0568	*sagG*	streptolysin S export ATP-binding protein	1.84	2.00E-05
Spy_0569	*sagH*	streptolysin S export transmembrane protein	2.02	2.57E-07
Spy_0570	*sagI*	streptolysin S export transmembrane protein	1.83	3.61E-05
Spy_0648	*rpsP*	30S ribosomal protein S16	-1.14	1.34E-05
Spy_0651	-	cell surface protein	-1.81	8.35E-07
Spy_0652	-	hypothetical protein	-1.53	8.76E-02
Spy_0665	-	transposase	3.33	1.60E-02
Spy_0666	-	hypothetical protein	5.21	4.24E-03
Spy_0667	*speC*	pyrogenic exotoxin type C	9.06	1.72E-09
Spy_0668	*mac*	IgG-degrading protease	10.01	1.45E-15
Spy_0707	-	cystine transporter permease	1.08	5.39E-04
Spy_0777	-	hypothetical protein	2.93	3.22E-36
Spy_0858	*xpt*	xanthine phosphoribosyltransferase	-1.59	4.41E-02
Spy_0859	-	xanthine permease	-1.00	3.12E-02
Spy_0978	-	Na(+)-linked D-alanine glycine permease	-1.06	4.29E-02
Spy_0996	*speA2*	enterotoxin	2.54	9.97E-08
Spy_1066	*amyB*	neopullulanase/cyclomaltodextrinase/maltogenic alpha-amylase	1.01	2.06E-03
Spy_1079	-	PTS system cellobiose-specific transporter subunit IIC	1.47	7.37E-02
Spy_1114	-	hypothetical protein	1.53	1.27E-08
Spy_1139	*nagB*	glucosamine-6-phosphate isomerase	-2.37	1.15E-02
Spy_1142	-	hypothetical protein	1.37	8.45E-05
Spy_1144	-	hypothetical protein	1.95	5.28E-05
Spy_1169	*spd3*	DNase	1.23	4.58E-03
Spy_1170	-	hypothetical protein	1.15	5.78E-03
Spy_1237	*artP*	arginine transport ATP-binding protein	-1.58	1.60E-03
Spy_1238	*artQ*	arginine transporter permease	-1.16	7.37E-02
Spy_1270	*arcC*	carbamate kinase	1.06	4.33E-02
Spy_1273	*arcB*	ornithine carbamoyltransferase	1.12	3.66E-03
Spy_1274	-	acetyltransferase	1.28	8.20E-04
Spy_1275	*arcA*	arginine deiminase	1.12	2.42E-03
Spy_1286	-	DNA polymerase	2.00	2.55E-06

Spy_1287	-	hypothetical protein	1.13	9.47E-02
Spy_1288	-	hypothetical protein	1.52	4.94E-03
Spy_1289	-	hypothetical protein	2.39	2.90E-08
Spy_1290	-	hypothetical protein	2.23	6.65E-04
Spy_1291	-	ATP-dependent RNA helicase	3.50	8.47E-12
Spy_1401	-	PTS system galactose-specific transporter subunit IIA	1.11	2.20E-05
Spy_1407	-	esterase	1.43	7.08E-02
Spy_1415	*sdaD2*	phage-encoded DNase	3.28	4.44E-15
Spy_1472	*hit*	bis(5'-nucleosyl)-tetraphosphatase (asymmetrical)	1.52	1.69E-09
Spy_1473	-	hypothetical protein	1.34	3.61E-05
Spy_1477	-	guanine-hypoxanthine permease	-1.87	3.09E-07
Spy_1492	*fabK*	enoyl-ACP reductase	-1.01	2.80E-03
Spy_1493	*acpP*	acyl carrier protein	-1.03	8.05E-05
Spy_1517	-	lantibiotic transport permease	1.48	6.82E-03
Spy_1528	-	ferrichrome-binding protein	-1.83	5.35E-02
Spy_1531	*isp2*	hypothetical protein	1.99	4.27E-29
Spy_1540	*endoS*	endo-beta-N-acetylglucosaminidase F2	-1.29	9.05E-03
Spy_1542	*scrA*	PTS system sucrose-specific transporter subunit IIABC	-1.04	4.33E-02
Spy_1556	-	hypothetical protein	3.89	3.94E-14
Spy_1557	*mutY*	A/G-specific adenine glycosylase	2.05	1.59E-16
Spy_1573	*glpF.2*	glycerol uptake facilitator protein	-1.06	1.21E-04
Spy_1646	*prsI*	30S ribosomal protein S9	-1.13	8.34E-07
Spy_1647	*rplM*	50S ribosomal protein L13	-1.08	1.16E-04
Spy_1684	*ska*	streptokinase	4.32	7.70E-65
Spy_1687	*sclA*	collagen-like surface protein	7.75	9.53E-07
Spy_1689	-	hypothetical protein	10.35	2.00E-12
Spy_1704	*dppA*	dipeptide-binding protein	-1.93	5.39E-18
Spy_1705	*dppB*	dipeptide transporter permease	-1.39	2.42E-03
Spy_1706	*dppC*	dipeptide transporter permease	-1.77	1.21E-04
Spy_1707	*dppD*	dipeptide transport ATP-binding protein	-1.84	1.23E-04
Spy_1708	*dppE*	dipeptide transport ATP-binding protein	-1.80	4.35E-04
Spy_1709	-	hypothetical protein	1.13	8.35E-07
Spy_1714	-	cell surface protein	1.53	4.08E-15
Spy_1715	*scpA*	C5A peptidase	1.76	6.10E-12
Spy_1718	*sic1.01*	inhibitor of complement protein	2.99	1.13E-56
Spy_1731	-	hypothetical protein	2.29	3.88E-03
Spy_1732	*prsA*	foldase PrsA	-1.08	2.10E-04
Spy_1733	-	hypothetical protein	3.69	2.10E-04
Spy_1738	*spd*	phage-associated deoxyribonuclease	2.00	8.21E-13
Spy_1782	*pepO*	neutral endopeptidase	1.56	1.00E-15

Spy_1839	-	MutT/nudix family protein	-1.15	6.48E-02
Spy_1851	*hasA*	hyaluronan synthase	6.41	3.57E-89
Spy_1852	*hasB*	UDP-glucose 6-dehydrogenase	6.16	4.86E-110
Spy_1853	*hasC*	UTP-glucose-1-phosphate uridylyltransferase	6.10	7.34E-96
Shaded genes indicate a member of a putative operon.				
*Gene locus numbers refer to MGAS5005 genome.				
