# Supplementary material for: Identification of Group A Streptococcus Genes Directly Regulated by CsrRS and Novel Intermediate Regulators
Source: mBio. 2021 Jul 13;12(4):e01642-21. doi: 10.1128/mBio.01642-21 (PMC8406183; doi:10.1128/mBio.01642-21)
Supplement: TABLE S2 [file mbio.01642-21-st002.pdf]

Table S2. List of genes included in NanoString studies.								
			Log2-fold change ΔcsrR vs. WT		Reason included in NanoString library			
Gene Locus*	Gene name	Gene product	RNAseq	NanoString	Additional assay (this study)			Previous study
Spy_0017	*sibA*	secreted protein	-1.04					(2, 3)
Spy_0029	*purD*	phosphoribosylamine-glycine ligase	-1.25		RNAseq			(2)
Spy_0034	-	transcription regulator	1.37	1.11	RNAseq	LL37 ChIP	Mg ChIP	
Spy_0083	*rpoB*	DNA-directed RNA polymerase subunit beta				LL37 ChIP	Mg ChIP	
Spy_0095	-	hypothetical protein						(3)
Spy_0106	*rofA*	transcriptional regulator				LL37 ChIP		(2, 14)
Spy_0113	-	transposase	7.17	7.63	RNAseq			
Spy_0115	-	hypothetical protein	9.45	10.21	RNAseq	LL37 ChIP	Mg ChIP	(2, 3, 14)
Spy_0124	-	Putative regulatory protein						(3)
Spy_0131	*ntpA*	putative V-type Na ATPase alpha subunit	1.08		RNAseq			(3)
Spy_0139	*nga*	NAD glycohydrolase	3.12	3.06	RNAseq		Mg ChIP	(2, 14)
Spy_0140	*ifs*	hypothetical protein			RNAseq			(14)
Spy_0141	*slo*	streptolysin O	2.55	2.87	RNAseq	LL37 ChIP		(2, 14)
Spy_0142	-	hypothetical protein		2.22			Mg ChIP	(14)
Spy_0143	-	hypothetical protein	11.99	11.49	RNAseq	LL37 ChIP	Mg ChIP	(3)
Spy_0144	-	hypothetical protein	9.87	10.52	RNAseq	LL37 ChIP	Mg ChIP	(3, 14)
Spy_0145	-	hypothetical protein				LL37 ChIP	Mg ChIP	
Spy_0146	*metB*	cystathionine beta-lyase				LL37 ChIP	Mg ChIP	
Spy_0157	*opuAA*	glycine betaine transport ATP-binding protein	-1.89	-2.74	RNAseq			(2)
Spy_0158	*opuABC*	glycine betaine-binding protein/glycine betaine transporter permease			RNAseq			(2, 3)

Spy_0161	*perR*	ferric transport regulator protein						(2)
Spy_0180	-	murein endopeptidase		1.56	RNAseq			(2)
Spy_0182	*speG*	exotoxin type G				LL37 ChIP		
Spy_0186	-	transcription regulator	1.67	2.66	RNAseq	LL37 ChIP	Mg ChIP	(2)
Spy_0195	-	MarR family transcription regulator	-1.69	-1.71	RNAseq	LL37 ChIP		
Spy_0205	*fasC*	putative histidine kinase						(2)
Spy_0275	-	serine/threonine transporter SstT	-2.05	-1.67	RNAseq	LL37 ChIP	Mg ChIP	
Spy_0281	-	hypothetical protein		1.02			Mg ChIP	
Spy_0282	*csrR*	response regulator	-12.95	-8.69	RNAseq	LL37 ChIP	Mg ChIP	(2)
Spy_0341	*scpC/prtS*	IL-8 protease	2.68	4.58	RNAseq	LL37 ChIP	Mg ChIP	(2, 3, 14)
Spy_0351	*spyA*	C3 family ADP-ribosyltransferase	3.02	3.27	RNAseq		Mg ChIP	(2, 14)
Spy_0352	-	hypothetical protein	6.33	7.29	RNAseq		Mg ChIP	
Spy_0354	-	hypothetical protein	3.14	3.51	RNAseq		Mg ChIP	
Spy_0355	-	hypothetical protein	4.18	6.6	RNAseq	LL37 ChIP	Mg ChIP	
Spy_0356	*speJ*	pyrogenic exotoxin type J	1.84	2.16	RNAseq		Mg ChIP	
Spy_0357	-	hypothetical protein	2.42	2.35	RNAseq		Mg ChIP	
Spy_0402	-	hypothetical protein					Mg ChIP	
Spy_0419	-	permease				LL37 ChIP		(2)
Spy_0425	-	putative sugar transferase						(3)
Spy_0436	*vicK*	two-component sensor histidine kinase						(2)
Spy_0440	-	transcriptional regulator				LL37 ChIP		(2)
Spy_0444	-	hypothetical protein				LL37 ChIP	Mg ChIP	

Spy_0473	-	multidrug resistance protein B				LL37 ChIP		
Spy_0474	*licT*	BglG family transcription antiterminator				LL37 ChIP		
Spy_0475	-	beta-glucoside permease IIABC component						(3)
Spy_0476	*bglA*	beta-glucosidase						
Spy_0500	-	N-acetylmuramoyl-L-alanine amidase		1.07		LL37 ChIP	Mg ChIP	
Spy_0501	-	hypothetical protein				LL37 ChIP	Mg ChIP	
Spy_0529	-	(Fe-S)-binding protein				LL37 ChIP	Mg ChIP	
Spy_0545	*agaS*	putative tagatose-6-phosphate aldose/ketose isomerase						(2, 3)
Spy_0551	*rplS*	50S ribosomal protein L19	-1.77	-1.38	RNAseq			(2)
Spy_0561	*epf*	extracellular matrix binding protein	4.30	3.38	RNAseq	LL37 ChIP	Mg ChIP	
Spy_0562	*sagA*	streptolysin S			RNAseq	LL37 ChIP	Mg ChIP	(2, 3)
Spy_0563	*sagB*	streptolysin S biosynthesis protein	1.83	0.99	RNAseq			(2, 14)
Spy_0571	-	hypothetical protein					Mg ChIP	
Spy_0598	*mscL*	large-conductance mechanosensitive channel				LL37 ChIP		(2)
Spy_0622	-	methyltransferase				LL37 ChIP		(2)
Spy_0635	*rpmA*	50S ribosomal protein L27		-1.19				
Spy_0639	*pyrR*	putative pyrimidine regulatory protein		-1.79				(3)
Spy_0640	*pyrP*	putative uricil permease		-1.56				
Spy_0641	*pyrB*	putative aspartate transcarbamoylase				LL37 ChIP		(3)
Spy_0642	*carA*	putative carbamoylphosphate synthetase small subunit						(3)
Spy_0643	*carB*	putative carbamoylphosphate synthetase						(2, 3)

Spy_0648	*rpsP*	30S ribosomal protein S16	-1.14		RNAseq	LL37 ChIP		
Spy_0651	-	cell surface protein	-1.81	-1.01	RNAseq	LL37 ChIP	Mg ChIP	(2, 14)
Spy_0652	-	hypothetical protein	-1.53	-1.19	RNAseq			(3)
Spy_0668	*mac*	IgG-degrading protease	10.01	7.42	RNAseq	LL37 ChIP	Mg ChIP	(2, 3, 14)
Spy_0673	*papS*	tRNA CCA-pyrophosphorylase				LL37 ChIP		(2)
Spy_0701	*cpsY*	transcriptional regulator						(2)
Spy_0713	*bcaT*	branched-chain amino acid aminotransferase				LL37 ChIP	Mg ChIP	
Spy_0760	-	UDP-N-acetylmuramoylalanyl-D-glutamate--2,6-diaminopimelate ligase				LL37 ChIP		
Spy_0761	-	hypothetical protein				LL37 ChIP		
Spy_0777	-	hypothetical protein	2.93	3.34	RNAseq		Mg ChIP	(2)
Spy_0784	*yesN*	putative two-component sensor histitdine kinase				LL37 ChIP		(2, 3)
Spy_0790	*gabD*	putative succinic semialdehyde dehydrogenase						(3)
Spy_0804	*srtK*	putative histidine kinase						(2)
Spy_0809	*srtE*	conserved hypothetical protein - lantibiotic						(3)
Spy_0817	*dacA1*	D-alanyl-D-alanine carboxypeptidase				LL37 ChIP		
Spy_0831	*dpiB*	sensor kinase				LL37 ChIP		(2)
Spy_0858	*xpt*	xanthine phosphoribosyltransferase	-1.59	-0.98	RNAseq			
Spy_0859	-	xanthine permease	-1.00	-0.98	RNAseq	LL37 ChIP		
Spy_0874	*gyrA*	DNA gyrase subunit A						
Spy_0913	-	putative integrase/recombinase						(3)
Spy_0947	*ciaH*	sensor protein				LL37 ChIP		(2)

Spy_0978	-	Na(+)-linked D-alanine glycine permease	-1.06		RNAseq	LL37 ChIP		(2)
Spy_0981	*cfa*	cAMP factor				LL37 ChIP		(2)
Spy_0996	*speA2*	enterotoxin	2.54	2.35	RNAseq		Mg ChIP	(14)
Spy_1048	-	phage protein				LL37 ChIP		
Spy_1079	-	PTS system cellobiose-specific transporter subunit IIC	1.47		RNAseq			
Spy_1094	-	hypothetical protein						(3)
Spy_1106	*grab*	protein G-related alpha 2M-binding protein		2.79		LL37 ChIP	Mg ChIP	(2, 3)
Spy_1114	-	hypothetical protein	1.53	1.75	RNAseq	LL37 ChIP		
Spy_1119	*gapN*	NADP-dependent glyceraldehyde-3-phosphate dehydrogenase				LL37 ChIP		(2)
Spy_1137	-	putative transcription factor						(2)
Spy_1139	*nagB*	glucosamine-6-phosphate isomerase	-2.37		RNAseq	LL37 ChIP	Mg ChIP	
Spy_1152	-	putative cation (K+) transport protein						
Spy_1155	*prfC*	peptide chain release factor 3				LL37 ChIP		(2)
Spy_1169	*spd3*	DNase	1.23	1.07	RNAseq	LL37 ChIP		(2, 14)
Spy_1170	-	hypothetical protein	1.15	1.03	RNAseq			
Spy_1171	-	conserved hypothetical protein, phage associated						(3)
Spy_1200	-	conserved hypothetical protein, phage associated						(3)
Spy_1237	*artP*	arginine transport ATP-binding protein			RNAseq			
Spy_1238	*artQ*	arginine transporter permease	-1.16	-1.35	RNAseq			
Spy_1267	*ktdB*	putative 3-deoxy-D-manno-octulosonic-acid transferase						(3)

Spy_1269	*asnA*	putative asparagine synthase A				LL37 ChIP		(3)
Spy_1274	-	acetyltransferase	1.28	1.74	RNAseq			
Spy_1284	*ccdA*	putative cytochrome C-type biogenesis protein						(3)
Spy_1290	-	hypothetical protein	2.23	1.44	RNAseq	LL37 ChIP		
Spy_1291	-	ATP-dependent RNA helicase	3.50	2.61	RNAseq		Mg ChIP	
Spy_1305	*lytR*	putative two-component sensor response regulator						(2)
Spy_1320	-	hypothetical protein rexA						(3)
Spy_1329	*cysM*	putative O-acetylserine lyase		-1.13				(3)
Spy_1386	-	hypothetical protein		-1.36			Mg ChIP	(2)
Spy_1401	-	PTS system galactose-specific transporter subunit IIA	1.11	2.26	RNAseq			(3)
Spy_1407	-	esterase	1.43	2.64	RNAseq		Mg ChIP	(14)
Spy_1411	-	hypothetical protein						(2, 3)
Spy_1415	*sdaD2*	phage-encoded DNase	3.28	3.54	RNAseq			(14)
Spy_1472	*hit*	bis(5'-nucleosyl)-tetraphosphatase (asymmetrical)			RNAseq			
Spy_1473	-	hypothetical protein	1.34	1.14	RNAseq			
Spy_1477	-	guanine-hypoxanthine permease	-1.87	-1.99	RNAseq			(2)
Spy_1479	*manL*	PTS system mannose-specific transporter subunit IIAB				LL37 ChIP		(2)
Spy_1492	*fabK*	enoyl-ACP reductase	-1.01		RNAseq		Mg ChIP	(2)
Spy_1499	*grpE*	putative Hsp-70 cofactor						(2, 3)
Spy_1503	-	phosphoglycerate mutase				LL37 ChIP		
Spy_1504	-	hypothetical protein				LL37 ChIP		(2)
Spy_1512	*codY*	transcriptional repressor CodY				LL37 ChIP	Mg ChIP	

Spy_1513	-	aminotransferase				LL37 ChIP		
Spy_1514	-	universal stress protein				LL37 ChIP		(2)
Spy_1524	-	putative ABC transporter (ATP-binding protein)						(2, 3)
Spy_1525	-	putative ABC transporter (ATP-binding protein)						(2, 3)
Spy_1526		putative ABC transporter (ATP-binding protein)						(3)
Spy_1527		putative ABC transporter (permease)						
Spy_1528	-	ferrichrome-binding protein	-1.83		RNAseq			(2)
Spy_1531	*isp2*	hypothetical protein	1.99	2.12	RNAseq	LL37 ChIP		(2)
Spy_1540	*endoS*	endo-beta-N-acetylglucosaminidase F2	-1.29		RNAseq		Mg ChIP	(2)
Spy_1545	*nusB*	putative transcriptional terminator						(3)
Spy_1556	-	hypothetical protein	3.89	3.84	RNAseq	LL37 ChIP	Mg ChIP	
Spy_1557	*mutY*	A/G-specific adenine glycosylase			RNAseq	LL37 ChIP	Mg ChIP	(2)
Spy_1573	*glpF.2*	glycerol uptake facilitator protein	-1.06		RNAseq			
Spy_1578	-	Cro/CI family transcriptional regulator				LL37 ChIP	Mg ChIP	
Spy_1579	-	transcriptional regulator				LL37 ChIP	Mg ChIP	
Spy_1601	-	membrane protease					Mg ChIP	(2)
Spy_1607	*fba*	fructose-bisphosphate aldolase						(14)
Spy_1635	*lacD.2*	putative tagatose 1,6-diphosphate aldolase						(2, 3)
Spy_1636	*lacC.2*	putative galactose-6-phosphate isomerase (C subunit)						(2, 3)
Spy_1637	*lacB.2*	putative galactose-6-phosphate isomerase (B subunit)						(2, 3)

Spy_1638	*lacA*	galactose-6-phosphate isomerase subunit LacA				LL37 ChIP		(2, 3)
Spy_1646	*prsI*	30S ribosomal protein S9			RNAseq			
Spy_1647	*rplM*	50S ribosomal protein L13	-1.08		RNAseq			
Spy_1649	-	Putative transcription regulator						(3)
Spy_1673	*proS*	prolyl-tRNA synthetase						
Spy_1680	*pulA*	Pullulanase						
Spy_1684	*ska*	streptokinase	4.32	4.29	RNAseq	LL37 ChIP	Mg ChIP	(2, 3)
Spy_1687	*sclA*	collagen-like surface protein	7.75		RNAseq			(14)
Spy_1702	*smeZ*	mitogenic exotoxin Z					Mg ChIP	(2)
Spy_1704	*dppA*	dipeptide-binding protein	-1.93	-2.52	RNAseq	LL37 ChIP	Mg ChIP	(2, 14)
Spy_1709	-	hypothetical protein	1.13		RNAseq			
Spy_1711	*lmb*	putative laminin adhesion						(2)
Spy_1714	-	cell surface protein	1.53	1.73	RNAseq			(2)
Spy_1715	*scpA*	C5A peptidase	1.76	1.92	RNAseq		Mg ChIP	(2, 14)
Spy_1718	*sic1.01*	inhibitor of complement protein	2.99	3.79	RNAseq	LL37 ChIP	Mg ChIP	(2, 14)
Spy_1719	*emm1.0*	M protein					Mg ChIP	(2, 14)
Spy_1720	*mga*	trans-acting positive regulator				LL37 ChIP	Mg ChIP	(2, 14)
Spy_1724	*irr*	putative two-component response regulator						(2)
Spy_1731	-	hypothetical protein	2.29	2.45	RNAseq			
Spy_1732	*prsA*	foldase PrsA	-1.08		RNAseq			(2)
Spy_1733	-	hypothetical protein	3.69	1.05	RNAseq			
Spy_1735	*speB*	exotoxin B		1.18		LL37 ChIP	Mg ChIP	(2)
Spy_1737	*ropB*	transcriptional regulator					Mg ChIP	(2)
Spy_1738	*spd*	phage-associated deoxyribonuclease	2.00	2.25	RNAseq	LL37 ChIP	Mg ChIP	(2, 3)
Spy_1757	-	hypothetical protein				LL37 ChIP		(2)
Spy_1758	-	dipeptidase B		1.81		LL37 ChIP		(2)

Spy_1768	*ahpC*	peroxiredoxin reductase (NAD(P)H)				LL37 ChIP	Mg ChIP	(2)
Spy_1782	*pepO*	neutral endopeptidase	1.56	1.52	RNAseq		Mg ChIP	
Spy_1794	-	hypothetical protein					Mg ChIP	
Spy_1799	*recA*	recombination protein				LL37 ChIP	Mg ChIP	
Spy_1825	-	PadR family transcriptional regulator					Mg ChIP	(2)
Spy_1839	-	MutT/nudix family protein	-1.15		RNAseq			(2)
Spy_1842	*sdhA*	putative L-serine dehydratase alpha subunit						(3)
Spy_1843	-	transglycosylase				LL37 ChIP	Mg ChIP	(3)
Spy_1851	*hasA*	hyaluronan synthase	6.41	7.18	RNAseq	LL37 ChIP	Mg ChIP	(2, 3, 14)
Spy_1857	*guaB*	inosine monophosphate dehydrogenase						(3)
Shaded boxes indicate significant transcript change identified by NanoString, but not by RNAseq.								
*Gene locus numbers refer to MGAS5005 genome.								
