# Supplementary material for: Identification of Group A Streptococcus Genes Directly Regulated by CsrRS and Novel Intermediate Regulators
Source: mBio. 2021 Jul 13;12(4):e01642-21. doi: 10.1128/mBio.01642-21 (PMC8406183; doi:10.1128/mBio.01642-21)
Supplement: TABLE S3 [file mbio.01642-21-st003.pdf]

Table S3. NanoString analysis of genes differentially transcribed in a CsrS H280A mutant or during growth of wild type strain 854 in the presence of 300 nM LL-37				
**Gene locus***	**Gene Name**	**Gene Product**	**Log2-fold change H280A vs. WT**	**Log2-fold change LL37 vs. unsupplemented**
Spy_1106	*grab*	protein G-related alpha 2M-binding protein	-4.17	-1.3
Spy_0146	*metB*	cystathionine beta-lyase	-3.8	-1.07
Spy_0545	*agaS*	putative tagatose-6-phosphate aldose/ketose isomerase	-2.92	-1.04
Spy_1152	-	putative cation (K+) transport protein	-2.53	-0.68
Spy_0598	*mscL*	large-conductance mechanosensitive channel	-2.3	-0.72
Spy_1737	*ropB*	transcription regulator	-2.06	-0.5
Spy_0563	*sagB*	Streptolysin S synthesis protein	-2.01	0.06
Spy_0562	*sagA*	streptolysin S	-1.92	-0.56
Spy_0195	-	MarR family transcription regulator	-1.85	0.4
Spy_0529	-	(Fe-S)-binding protein	-1.6	-0.71
Spy_1386	-	hypothetical protein	-1.42	-0.79
Spy_0981	*cfa*	cAMP factor	-1.35	-0.61
Spy_0473	-	multidrug resistance protein B	-1.24	-0.18
Spy_0639	*pyrR*	putative pyrimidine regulatory protein	-1.22	-0.25
Spy_0157	*opuAA*	glycine betaine transport ATP-binding protein	-1.22	0.09
Spy_1169	*spd3*	DNase	-1.18	1.19
Spy_1735	*speB*	cysteine protease SpeB	-1.08	0.29
Spy_1269	*asnA*	putative asparagine synthase A	-1.05	-0.21
Spy_1170	-	hypothetical protein	-1.05	1.11
Spy_0622	-	methyltransferase	-0.99	-0.49
Spy_0551	*rplS*	50S ribosomal protein L19	-0.98	-0.43
Spy_1473	-	hypothetical protein	1.03	0.47
Spy_1579	-	transcription regulator	1.04	0.41
Spy_0474	*licT*	BglG family transcription antiterminator	1.07	0.42
Spy_0180	-	murein endopeptidase	1.22	0.56
Spy_1525	-	putative ABC transporter (ATP-binding protein)	1.27	0.54
Spy_0475	-	beta-glucoside permease IIABC component	1.33	0.47

Spy_0476	*bglA*	beta-glucosidase	1.36	0.34
Spy_0034	-	transcription regulator	1.45	1.3
Spy_1782	*pepO*	neutral endopeptidase	1.48	0.59
Spy_1526	-	putative ABC transporter (ATP-binding protein)	1.53	0.72
Spy_1557	*mutY*	A/G-specific adenine glycosylase	1.64	0.87
Spy_1291	-	ATP-dependent RNA helicase	1.64	1.34
Spy_1684	*ska*	streptokinase	1.64	1.57
Spy_1528	-	ferrichrome-binding protein	1.68	0.74
Spy_1714	-	cell surface protein	1.87	1.24
Spy_1731	-	hypothetical protein	2.1	0.8
Spy_1715	*scpA*	C5A peptidase	2.17	1.39
Spy_0777	-	hypothetical protein	2.18	0.22
Spy_0356	*speJ*	pyrogenic exotoxin type J	2.23	0.81
Spy_0357	-	hypothetical protein	2.46	1.01
Spy_0186	-	transcription regulator	2.55	1.25
Spy_0142	-	hypothetical protein	2.66	1.87
Spy_0354	-	hypothetical protein	3.04	0.96
Spy_1556	-	hypothetical protein	3.08	1.04
Spy_0996	*speA2*	enterotoxin	3.08	1.1
Spy_0141	*slo*	streptolysin O	3.11	2.17
Spy_1415	*sdaD2*	phage-encoded DNase	3.18	2.03
Spy_0140	*ifs*	NADase inhibitor	3.21	2.05
Spy_0351	*spyA*	C3 family ADP-ribosyltransferase	3.25	1.89
Spy_0139	*nga*	NAD glycohydrolase	3.45	2.25
Spy_1718	*sic1.01*	inhibitor of complement protein	3.71	2.14
Spy_1407	-	esterase	4.82	2.38
Spy_0341	*scpC/prtS*	IL-8 protease	4.87	1.54
Spy_0113	-	transposase	5.1	1.65
Spy_0143	-	hypothetical protein	5.28	2.2
Spy_0668	*mac*	IgG-degrading protease	5.8	1.89
Spy_0352	-	hypothetical protein	5.91	2.99
Spy_0355	-	hypothetical protein	6.09	3.45
Spy_1851	*hasA*	hyaluronan synthase	6.31	3.13
Spy_0115	-	hypothetical protein	7.28	3.29
Spy_0144	-	hypothetical protein	7.62	4.1

Genes are listed for which a change in expression of 1 log2-fold or more, up or down, was observed in mutant strain 854 CsrS H280A compared to wild type 854.

Green or red shading is proportional to the degree of down- or up-regulation, respectively, in the mutant compared to wild type or during wild type growth in LL-37 compared to unsupplemented broth.

*Gene locus numbers refer to MGAS5005 genome.
