# Supplementary material for: Identification of Group A Streptococcus Genes Directly Regulated by CsrRS and Novel Intermediate Regulators
Source: mBio. 2021 Jul 13;12(4):e01642-21. doi: 10.1128/mBio.01642-21 (PMC8406183; doi:10.1128/mBio.01642-21)
Supplement: TABLE S4 [file mbio.01642-21-st004.pdf]

**Table S4. NanoString analysis of genes differentially transcribed in a CsrS T284A mutant or during growth of wild type strain 854 in the presence of 15 mM MgCl2**				
**Gene locus***	**Gene Name**	**Gene Product**	**Log2-fold change T284A vs. WT**	**Log2-fold change Mg vs. unsupplemented**
Spy_0140	*ifs*	NADase inhibitor	-4.02	-2.82
Spy_0139	*nga*	NAD glycohydrolase	-3.94	-2.77
Spy_0141	*slo*	streptolysin O	-3.77	-2.89
Spy_1170	-	hypothetical protein	-3.31	-1.71
Spy_0563	*sagB*	Streptolysin S synthesis protein	-3.3	-1.81
Spy_1169	*spd3*	DNase	-3.3	-1.64
Spy_1684	*ska*	streptokinase	-3.28	-1.84
Spy_1714	-	cell surface protein	-3.12	-1.4
Spy_1718	*sic1.01*	inhibitor of complement protein	-3.02	-1.54
Spy_0562	*sagA*	streptolysin S	-2.9	-1.63
Spy_1715	*scpA*	C5A peptidase	-2.79	-1.31
Spy_1719	*emm1.0*	M protein	-2.3	-0.64
Spy_1415	*sdaD2*	phage-encoded DNase	-2.28	-1.8
Spy_1687	*sclA*	hypothetical protein	-2.19	-1.27
Spy_0351	*spyA*	C3 family ADP-ribosyltransferase	-2.1	-1.37
Spy_0561	*epf*	extracellular matrix binding protein	-1.95	-1.44
Spy_1731	-	hypothetical protein	-1.94	-0.5
Spy_1704	*dppA*	dipeptide-binding protein	-1.82	-0.75
Spy_0571	-	hypothetical protein	-1.62	-0.93
Spy_0635	*rpmA*	50S ribosomal protein L27	-1.6	-0.65
Spy_0142	-	hypothetical protein	-1.56	-1.37
Spy_0529	-	(Fe-S)-binding protein	-1.55	-0.6
Spy_0622	-	methyltransferase	-1.53	-0.78
Spy_0639	*pyrR*	putative pyrimidine regulatory protein	-1.49	-0.33
Spy_0551	*rplS*	50S ribosomal protein L19	-1.48	-0.58
Spy_1386	-	hypothetical protein	-1.45	-0.98
Spy_1527	-	putative ABC transporter (permease)	-1.33	-0.77
Spy_0545	*agaS*	putative tagatose-6-phosphate aldose/ketose isomerase	-1.22	-0.6
Spy_0195	-	MarR family transcription regulator	-1.21	-0.18
Spy_1556	-	hypothetical protein	-1.2	-0.44
Spy_0598	*mscL*	large-conductance mechanosensitive channel	-1.16	-0.64
Spy_0143	-	hypothetical protein	-1.11	-0.85
Spy_1290	-	hypothetical protein	-1.07	-0.79
Spy_0144	-	hypothetical protein	-1.03	-1.04

Spy_1782	*pepO*	neutral endopeptidase	-1.02	-0.64
Spy_1557	*mutY*	A/G-specific adenine glycosylase	-1.02	-0.31
Spy_1407	-	esterase	-1.01	-0.64
Spy_1171	-	conserved hypothetical protein, phage associated	-0.99	-0.91
Spy_1738	*spd/speMF*	phage-associated deoxyribonuclease	-0.98	-0.72
Spy_1578	-	Cro/CI family transcription regulator	-0.75	-1
Spy_1732	*prsA*	foldase PrsA	-0.38	-1.31
Spy_0157	*opuAA*	glycine betaine transport ATP-binding protein	0.32	1.15
Spy_0158	*opuABC*	glycine betaine-binding protein/glycine betaine transporter permease	0.38	1.04
Spy_1139	*nagB*	glucosamine-6-phosphate isomerase	0.5	1.36
Spy_1137	-	putative transcription factor	0.6	1.06
Spy_1499	*grpE*	putative Hsp-70 cofactor	0.76	1.05
Spy_1733	-	hypothetical protein	0.97	-0.46
Spy_0476	*bglA*	beta-glucosidase	0.99	0.52
Spy_1711	*lmb*	putative laminin adhesion	1	0.93
Spy_0981	*cfa*	cAMP factor	1.05	0.59
Spy_0029	*purD*	phosphoribosylamine-glycine ligase	1.08	0.7
Spy_1637	*lacB.2*	putative galactose-6-phosphate isomerase (B subunit)	1.15	1.28
Spy_0475	-	beta-glucoside permease IIABC component	1.2	0.62
Spy_1477	-	guanine-hypoxanthine permease	1.22	0.56
Spy_1735	*speB*	cysteine protease SpeB	1.27	-0.32
Spy_1636	*lacC.2*	putative galactose-6-phosphate isomerase (C subunit)	1.29	1.09
Spy_0858	*xpt*	xanthine phosphoribosyltransferase	1.3	0.94
Spy_0146	*metB*	cystathionine beta-lyase	1.36	0.83
Spy_1638	*lacA*	galactose-6-phosphate isomerase subunit LacA	1.42	1.09
Spy_0859	-	xanthine permease	1.73	0.84
Spy_1401	-	putative PTS system, enzyme IIA component	1.74	0.72
Spy_1106	*grab*	protein G-related alpha 2M-binding protein	2.21	1.72

Genes are listed for which a change in expression of 1 log2-fold or more, up or down, was observed in mutant strain 854 CsrS T284A compared to wild type 854.

Green or red shading is proportional to the degree of down- or up-regulation, respectively, in the mutant compared to wild type or during wild type growth in supplemental magnesium compared to unsupplemented broth.
*Gene locus numbers refer to MGAS5005 genome.
