# Supplementary material for: Identification of Group A Streptococcus Genes Directly Regulated by CsrRS and Novel Intermediate Regulators
Source: mBio. 2021 Jul 13;12(4):e01642-21. doi: 10.1128/mBio.01642-21 (PMC8406183; doi:10.1128/mBio.01642-21)
Supplement: TABLE S5 [file mbio.01642-21-st005.pdf]

Table S5. ChIP seq analysis of CsrR binding in GAS strain 854 wild type grown in 15 mM MgCl2					
**Gene Locus***	**Gene name**	**Gene product**	**Fold enrichment**	**Peak Start**	**Peak End**
Spy_0001	*dnaA*	chromosome replication initiator DnaA	3.3	631	1005
Spy_0010	-	beta-lactamase	4.94	9523	9761
Spy_0014	-	amino acid permease	6.73	14816	15181
Spy_0034	-	transcription regulator	6	53024	53402
Spy_0035	*ruvB*	Holliday junction DNA helicase RuvB	3.6	54156	54440
Spy_0083	*rpoB*	DNA-directed RNA polymerase subunit beta	9.55	93929	94181
Spy_0115	-	hypothetical protein	12.56	126543	126848
Spy_0135	-	hypothetical protein	4.41	145308	145550
Spy_0139	*nga*	NAD glycohydrolase	3.54	149942	150259
Spy_0142	-	hypothetical protein	5.1	153636	154062
Spy_0143	-	hypothetical protein	27.59	154683	155221
Spy_0144	-	hypothetical protein	39.55	155289	155652
Spy_0145/0146	*-/metB*	hypothetical protein/cystathionine beta-lyase	6.83	155877	156343
Spy_0148	*ulaA*	PTS system ascorbate-specific transporter subunit IIC	4.82	160327	160523
Spy_0160	-	CoA binding protein	5.49	174694	174913
Spy_0176	-	hypothetical protein	3.23	186400	186684
Spy_0182	*speG*	exotoxin type G	4.82	190616	190848
Spy_0186	-	transcription regulator	4.46	195406	195604
Spy_0247	-	D-alanyl-D-alanine carboxypeptidase	3.17	256680	256860
Spy_0248/0249	*dacA2*	D-alanyl-D-alanine carboxypeptidase	6.31	257175	257494
Spy_0268	-	hypothetical protein	4.62	278480	278711
Spy_0275	-	serine/threonine transporter SstT	12.26	285303	285620
Spy_0281	-	hypothetical protein	8.5	291445	291740
Spy_0282	*csrR*	response regulator	4.03	291982	293049
Spy_0283	*csrS*	transmembrane histidine kinase	2.36	293243	293434
Spy_0340	*lctO*	L-lactate oxidase	2.54	343382	343600
Spy_0341	*scpC/prtS*	IL-8 protease	15.24	344389	344790
Spy_0351	*spyA*	C3 family ADP-ribosyltransferase	18.49	358443	359060
Spy_0354	-	hypothetical protein	16.25	360572	360879
Spy_0355	-	hypothetical protein	9.1	361179	361687
Spy_0356	*speJ*	exotoxin type J	7.88	362530	362744
Spy_0357	-	hypothetical protein	5.88	516685	516882

Spy_0401/402	*-/-*	hypothetical protein/hypothetical protein	13.2	396371	396642
Spy_0416	-	glutaminyl-peptide cyclotransferase	2.89	409152	409391
Spy_0444	-	hypothetical protein	9.12	439504	439806
Spy_0444	-	hypothetical protein	3.67	440475	440708
Spy_0495	*lysS*	lysyl-tRNA synthetase	3.76	482215	482428
Spy_0500/0501	-	N-acetylmuramoyl-L-alanine amidase	11.72	485868	486243
Spy_0529	-	(Fe-S)-binding protein	5.88	363494	363748
Spy_0530	*prfB*	peptide chain release factor 2	2.8	517924	518046
Spy_0561	*epf*	extracellular matrix binding protein	10.77	552284	552593
Spy_0562	*sagA*	streptolysin S	4.97	1826812	1827163
Spy_0571	-	hypothetical protein	9.91	567845	568105
Spy_0607	*rgpEc*	glycosyltransferase	3.01	609691	609884
Spy_0651	-	cell surface protein	4.23	657909	658113
Spy_0657	*trxB*	thioredoxin reductase	4.82	662155	662583
Spy_0668	*mac*	IgG-degrading protease	9.46	672738	673011
Spy_0692	*engB*	ribosome biogenesis GTP-binding protein YsxC	4.15	694349	694549
Spy_0713	*bcaT*	branched-chain amino acid aminotransferase	6.96	717766	718011
Spy_0715	*rpsA*	30S ribosomal protein S1	2.89	719297	719595
Spy_0755	*acoL*	dihydrolipoamide dehydrogenase	3.75	757723	757902
Spy_0769	-	hypothetical protein	3.74	772792	772928
Spy_0777	-	hypothetical protein	51.81	782066	782414
Spy_0853	-	short chain dehydrogenase	3.16	843924	844075
Spy_0915	*ffh*	signal recognition particle subunit FFH/SRP54	2.47	902171	902289
Spy_0963	-	hypothetical protein	2.63	954063	954279
Spy_0970	-	GntR family transcription regulator	5.94	958812	959015
Spy_0981	*cfa*	cAMP factor	4.93	968980	969411
Spy_0996	*speA2*	enterotoxin	7.13	984116	984301
Spy_1105	-	ribosomal-protein-alanine acetyltransferase	5.62	1079015	1079193
Spy_1106	*grab*	protein G-related alpha 2M-binding protein	4.59	1079910	1080180
Spy_1129	-	CAAX amino protease	3.19	1105592	1105745
Spy_1132	*alaS*	alanyl-tRNA synthetase	4.49	1109602	1109726
Spy_1136	*pepB*	oligoendopeptidase F	2.4	1113833	1113967
Spy_1139	*nagB*	glucosamine-6-phosphate isomerase	4.72	1117448	1117620

Spy_1144	-	hypothetical protein	13.08	1120830	1121304
Spy_1146	*holA*	DNA polymerase III subunit delta	2.07	1123132	1123272
Spy_1148	-	competence protein ComE	3	1125813	1125992
Spy_1154	*deaD*	ATP-dependent RNA helicase	6.06	1132350	1132567
Spy_1260	-	prepilin peptidase	3.58	1216487	1216705
Spy_1280	-	two-component sensor kinase	2.3	1236536	1236719
Spy_1291	-	ATP-dependent RNA helicase	6.3	1248013	1248376
Spy_1307	-	hypothetical protein	2.81	1265578	1265750
Spy_1322	-	hypothetical protein	7.35	1284786	1285531
Spy_1341	*gmk*	guanylate kinase	3.53	1308366	1308588
Spy_1363	-	amino acid ABC transporter permease	2.59	1333102	1333273
Spy_1386	-	hypothetical protein	10.07	1360412	1360627
Spy_1389	-	sodium-dependent phosphate transporter	2.49	1364847	1364998
Spy_1390	-	hypothetical protein	3.94	1365414	1365625
Spy_1407	-	esterase	4.39	1377880	1378165
Spy_1415	*sdaD2*	phage-encoded DNase	4.85	1385591	1385805
Spy_1429	-	phage protein	2.33	1401865	1401974
Spy_1439	-	portal protein	3.11	1409157	1409429
Spy_1492	*fabK*	enoyl-ACP reductase	2.93	1448608	1448880
Spy_1512	*codY*	transcription repressor CodY	12.53	1465559	1465947
Spy_1518	-	transporter	3.52	1472269	1472629
Spy_1539	*scrK*	fructokinase	6.7	1498295	1498524
Spy_1540	*endoS*	endo-beta-N-acetylglucosaminidase F2	4.05	1501481	1501706
Spy_1555	*rpsF*	30S ribosomal protein S6	5.35	1515630	1515871
Spy_1557	*mutY*	A/G-specific adenine glycosylase	15.59	1516072	1516398
Spy_1559	*trx*	thioredoxin	3.36	1518733	1518942
Spy_1578/1579	-/-	Cro/CI family transcription regulator/transcription regulator	10.2	1540204	1540629
Spy_1601	-	membrane protease	5.64	1559797	1560031
Spy_1684	*ska*	streptokinase	14.69	1643866	1644494
Spy_1689	-	hypothetical protein	14.04	1649601	1650004
Spy_1702	*smeZ*	mitogenic exotoxin Z	3.96	1662198	1662439
Spy_1704	*dppA*	dipeptide-binding protein	5.9	1663478	1663723
Spy_1708	*dppE*	dipeptide transport ATP-binding protein	6.28	1668630	1669091
Spy_1715	*scpA*	C5A peptidase	5.57	1678792	1679106
Spy_1718	*sic1.01*	inhibitor of complement protein	9.74	1681790	1682081
Spy_1718	*sic1.01*	inhibitor of complement protein	2.26	1682450	1683193
Spy_1719	*emm1.0*	M protein	6.25	1683443	1683762

Spy_1720	*mga*	trans-acting positive regulator	11.11	1685397	1686398
Spy_1731	-	hypothetical protein	7.39	1694539	1694992
Spy_1735	*speB*	cysteine protease SpeB	3.18	1698328	1698815
Spy_1737	*ropB*	transcription regulator	4.06	1699076	1699329
Spy_1738	*spd*	phage-associated deoxyribonuclease	3.36	1701007	1701211
Spy_1768	*ahpC*	peroxiredoxin reductase (NAD(P)H)	6.77	1728339	1728762
Spy_1782	*pepO*	neutral endopeptidase	2.67	1750153	1750292
Spy_1793	*nrdD*	anaerobic ribonucleoside triphosphate reductase	8.13	1762505	1762777
Spy_1794	-	hypothetical protein	11.03	1763914	1764247
Spy_1803	*lmrP*	multidrug resistance protein B	3.35	1771108	1771348
Spy_1812	-	hypothetical protein	2.77	1781755	1782467
Spy_1824/1825	*-/-*	mutT/nudix family protein/PadR family transcription regulator	2.71	1791827	1792020
Spy_1843	-	transglycosylase	6.99	1811167	1811557
Spy_1849	-	zinc protease	6.28	1816924	1817330
Spy_1849	-	zinc protease	2.28	1817437	1817633
Spy_1851	*hasA*	hyaluronan synthase	9.69	1818203	1819059
Spy_1856	-	glucose uptake protein	3	1824932	1825305
Spy_1857	*guaB*	inosine 5'-monophosphate dehydrogenase	4.97	559165	559575
Spy_1857	*guaB*	inosine 5'-monophosphate dehydrogenase	4.44	1827452	1827695
Spy_T0065	-	Asn tRNA	7.68	1833765	1834257

Enrichment is determined with respect to the mock control. Greater than 2-fold enrichment is considered significant.

Genes are considered to be associated with a ChIP-seq peak if the translation start site is within 100 bp upstream to 500 bp downstream of the position of maximal enrichment.

Genes are listed multiple times if more than one ChIP-Seq peak was present within 100 bp upstream to 500 bp downstream of the transcription start site.

Multiple genes are listed for a single peak if that peak is within 100 bp upstream to 500 bp downstream of more than one gene.

*Gene locus numbers refer to MGAS5005 genome.
