# Supplementary material for: Identification of Group A Streptococcus Genes Directly Regulated by CsrRS and Novel Intermediate Regulators
Source: mBio. 2021 Jul 13;12(4):e01642-21. doi: 10.1128/mBio.01642-21 (PMC8406183; doi:10.1128/mBio.01642-21)
Supplement: TABLE S6 [file mbio.01642-21-st006.pdf]

Table S6. ChIP seq analysis of CsrR binding in GAS strain 854 wild type grown in 300 nM LL-37					
Gene Locus*	Gene name	Gene product	Fold enrichment	Peak Start	Peak End
Spy_0001	*dnaA*	chromosome replication initiator DnaA	2.2	285289	285629
Spy_0003	-	hypothetical protein	3.84	552217	552580
Spy_0007	-	heat shock protein 15	2.96	1728335	1728800
Spy_0008	*divIC*	cell division protein	4.58	463509	463792
Spy_0010	-	beta-lactamase	3.76	1132318	1132589
Spy_0012	*tilS*	tRNA(Ile)-lysidine synthetase	2.32	93923	94187
Spy_0013	*ftsH*	cell division protein	5.44	439540	439828
Spy_0014	-	amino acid permease	3.64	654754	654977
Spy_0019	*recO*	DNA repair protein RecO	7.4	1663469	1663725
Spy_0024	*purF*	amidophosphoribosyltransferase	3.77	1017458	1017702
Spy_0032	-	hypothetical protein	5.61	292005	292729
Spy_0032	-	hypothetical protein	4.45	411775	412088
Spy_0034	-	transcription regulator	3.69	361139	361409
Spy_0034	-	transcription regulator	3.58	559334	559545
Spy_0034	-	transcription regulator	2.96	1221701	1221955
Spy_0036	-	protein tyrosine phosphatase	6.86	1762497	1762792
Spy_0038	-	acyltransferase	2.16	191034	191258
Spy_0040	*adhA*	alcohol dehydrogenase	4.26	1435324	1435538
Spy_0063	*rplO*	50S ribosomal protein L15	2.41	438932	439145
Spy_0080	-	bis(5'-nucleosyl)-tetraphosphatase (asymmetrical)	2.57	717764	718004
Spy_0082	*pbp1b*	multimodular transpeptidase-transglycosylase PBP 1B	3.34	600524	600735
Spy_0083	*rpoB*	DNA-directed RNA polymerase subunit beta	17.23	1643948	1644440
Spy_0105	*hslO*	heat shock protein 33	4.57	1120865	1121305
Spy_0106/107	*rofA*	transcription regulator	4.3	1119797	1120000
Spy_0115	-	hypothetical protein	3.31	765461	765697
Spy_0135	-	hypothetical protein	7.83	662372	662600
Spy_0141	*slo*	streptolysin O	3.27	1459082	1459271
Spy_0143	-	hypothetical protein	4.56	812322	812558
Spy_0144	-	hypothetical protein	3.96	145295	145559
Spy_0145/0146	*-/metB*	hypothetical protein/cystathionine beta-lyase	3.72	1293655	1293905
Spy_0148	*ulaA*	PTS system ascorbate-specific transporter subunit IIC	3.95	34036	34235
Spy_0163	-	3'-phosphoadenosine 5'-phosphosulfate sulfotransferase	2.92	968940	969180

Spy_0182	*speG*	exotoxin type G	9.12	672227	672425
Spy_0182	*speG*	exotoxin type G	5.84	1466913	1467161
Spy_0186	-	transcription regulator	4.38	516662	516909
Spy_0186	-	transcription regulator	4.18	55380	55611
Spy_0195	-	MarR family transcription regulator	3.59	674825	675052
Spy_0206	*fasA*	response regulator	2.35	1827449	1827711
Spy_0207/0208	*rnpA*	ribonuclease P	2.64	925664	925869
Spy_0239	*mecA*	adaptor protein	3.72	626187	626535
Spy_0240	-	undecaprenyl-phosphate alpha-N-acetylglucosaminephosphotransferase	4.25	937660	937757
Spy_0248/0249	*dacA2*	D-alanyl-D-alanine carboxypeptidase	6.08	1471694	1471940
Spy_0251	*oppC*	oligopeptide transporter permease	3.28	1817087	1817320
Spy_0263	*nadD*	nicotinic acid mononucleotide adenylyltransferase	3.03	1133925	1134138
Spy_0268	-	hypothetical protein	3.41	444827	444999
Spy_0270	-	ABC transporter substrate-binding protein	2.06	694331	694564
Spy_0275	-	serine/threonine transporter SstT	23.46	257257	257492
Spy_0282	*csrR*	response regulator	13.78	1095447	1095632
Spy_0282	*csrR*	response regulator	2.15	905808	905934
Spy_0288	*snf*	SWF/SNF family helicase	2.82	1171489	1171662
Spy_0289	-	hypothetical protein	5.12	1602771	1603070
Spy_0309	*scpB*	segregation and condensation protein B	5.61	191517	191764
Spy_0329	-	hypothetical protein	2.93	1490539	1490763
Spy_0330	*tmk*	thymidylate kinase	3.62	1811322	1811507
Spy_0332	-	tpl protein	4.27	394128	394269
Spy_0340	*lctO*	L-lactate oxidase	2.89	395542	395865
Spy_0341	*scpC/prtS*	IL-8 protease	3.34	440278	440432
Spy_0355	-	hypothetical protein	11.63	1554426	1554668
Spy_0392	-	hypothetical protein	5.72	317091	317322
Spy_0400	*silD*	hypothetical protein	5.71	50903	51151
Spy_0419	-	permease	12.35	12674	12925
Spy_0420	-	glucosyltransferase	3.55	1364814	1365033
Spy_0427	*thrS*	threonyl-tRNA synthetase	2.95	1081512	1081661
Spy_0440	-	transcription regulator	4.81	528425	528608
Spy_0440/0441	-	transcription regulator	2.63	495349	495559
Spy_0443	-	hypothetical protein	9.08	1682461	1683029

Spy_0444	-	hypothetical protein	16.41	1130064	1130247
Spy_0444	-	hypothetical protein	5.66	891423	891615
Spy_0446	-	hypothetical protein	4.79	887897	888075
Spy_0449	-	UDP-glucose 6-dehydrogenase	6.37	1685615	1685852
Spy_0450	*mefE*	macrolide-efflux protein	3.83	302118	302313
Spy_0463	-	hypothetical protein	2.62	485891	486188
Spy_0473/0474	-	multidrug resistance protein B	19.3	691227	691385
Spy_0500/0501	-	N-acetylmuramoyl-L-alanine amidase	5.09	1015828	1015945
Spy_0506	*ftsW*	cell division protein	3.33	1079913	1080091
Spy_0509	*tpiA*	triosephosphate isomerase	5.25	436192	436278
Spy_0524	*idnO*	gluconate 5-dehydrogenase	2.94	1465591	1465797
Spy_0529	-	(Fe-S)-binding protein	6.94	442340	442509
Spy_0530	*prfB*	peptide chain release factor 2	3.29	1245622	1245775
Spy_0539	-	hypothetical protein	5.3	660291	660458
Spy_0560	-	transcription regulator	3.23	1619652	1619900
Spy_0561	*epf*	extracellular matrix binding protein	22.42	1145931	1146273
Spy_0562	*sagA*	streptolysin S	10.61	9089	9265
Spy_0564	*sagC*	streptolysin S biosynthesis protein	3.98	1698585	1698782
Spy_0569	*sagH*	streptolysin S export transmembrane protein	2.61	116307	116558
Spy_0580	*atpG*	ATP synthase F0F1 subunit gamma	4.18	154829	155140
Spy_0597	*rpsU*	30S ribosomal protein S21	8.5	1026189	1026344
Spy_0598/0599	*mscL/dnaG*	large-conductance mechanosensitive channel/DNA primase	4.35	1764039	1764182
Spy_0605	*rgpCc*	polysaccharide export ABC transporter permease	2.8	50397	50599
Spy_0614	*pepT*	peptidase T	2.68	1089425	1089546
Spy_0620	*rpmI*	50S ribosomal protein L35	2.85	1473824	1474062
Spy_0622/0623	-	phosphoglycerol transferase	6.62	195464	195593
Spy_0636	-	LysR family transcription regulator	2.97	601016	601205
Spy_0641	*pyrB*	aspartate carbamoyltransferase	3.03	757707	757912
Spy_0644	-	periplasmic protein of efflux system	2.9	117043	117339
Spy_0648	*rpsP*	30S ribosomal protein S16	3.59	339168	339330
Spy_0651	-	cell surface protein	16.08	61132	61361

Spy_0654	-	TetR family transcription regulator	4.69	248143	248375
Spy_0657	*trxB*	thioredoxin reductase	8.1	1333086	1333281
Spy_0668	*mac*	IgG-degrading protease	7.17	1765799	1765983
Spy_0668	*mac*	IgG-degrading protease	3.59	579759	579959
Spy_0673	*papS*	tRNA CCA-pyrophosphorylase	6.83	823341	823513
Spy_0687/688	*mvaS.1*	hydroxymethylglutaryl-CoA synthase	4.99	195757	196018
Spy_0692	*engB*	ribosome biogenesis GTP-binding protein YsxC	6.12	1365396	1365626
Spy_0713	*bcaT*	branched-chain amino acid aminotransferase	8.83	1145072	1145254
Spy_0724	-	hypothetical protein	2.45	898721	898794
Spy_0755	*acoL*	dihydrolipoamide dehydrogenase	4.31	1409150	1409382
Spy_0760/0761	*-/-*	UDP-N-acetylmuramoylalanyl-D-glutamate--2,6-diaminopimelate ligase/hypotheical protein	8.17	1037465	1037533
Spy_0817	*dacA1*	D-alanyl-D-alanine carboxypeptidase	7.95	561267	561412
Spy_0830	*dpiA*	transcription regulatory protein	4.18	1635541	1635740
Spy_0831/0832	*dpiB/malP*	sensor kinase/malate-sodium symport	3.5	155425	155586
Spy_0834	-	Zn-dependent alcohol dehydrogenase and related dehydrogenase	2.09	160338	160501
Spy_0847	-	GTP pyrophosphokinase	3.47	1219922	1220098
Spy_0853	-	short chain dehydrogenase	2.96	3205	3396
Spy_0859	-	xanthine permease	3.26	445530	445848
Spy_0899	*citG*	GntR family transcription regulator	5.19	1718048	1718188
Spy_0903	*oadB*	oxaloacetate decarboxylase subunit beta	5.21	1035247	1035435
Spy_0911/0912	*-/-*	hypothetical protein/hypothetical protein	4.11	1457292	1457530
Spy_0915	*ffh*	signal recognition particle subunit FFH/SRP54	3.45	1818435	1818787
Spy_0918/0919	*-/guaA*	hypothetical protein/GMP synthase	5.93	40880	41072
Spy_0925	*rnhB*	anaerobic ribonucleoside-triphosphate reductase	3.59	1321057	1321149
Spy_0936	*dfp*	phosphopantothenoylcysteine decarboxylase	6.66	9568	9658

Spy_0947	*ciaH*	sensor protein	6.61	247368	247626
Spy_0963	-	hypothetical protein	3.33	155939	156350
Spy_0970	-	GntR family transcription regulator	3.7	959272	959388
Spy_0981	*cfa*	cAMP factor	7.17	53009	53246
Spy_1019	-	phage scaffold protein	2.59	1799797	1799955
Spy_1043	-	phage protein	4.98	1753949	1754149
Spy_1048	-	phage protein	15.45	1308382	1308546
Spy_1056	*malM*	4-alpha-glucanotransferase	4.55	14996	15225
Spy_1063	*malD*	maltodextrin transporter permease	3.82	337579	337791
Spy_1063	*malD*	maltodextrin transporter permease	2.64	672788	672988
Spy_1065	*amyA*	alpha-amylase	3.98	653996	654141
Spy_1106	*grab*	protein G-related alpha 2M-binding protein	4.95	913342	913403
Spy_1107	*murZ*	UDP-N-acetylglucosamine 1-carboxyvinyltransferase	5.38	200329	200538
Spy_1114	-	hypothetical protein	4.4	53966	54102
Spy_1119	*gapN*	NADP-dependent glyceraldehyde-3-phosphate dehydrogenase	6.02	413147	413347
Spy_1139	*nagB*	glucosamine-6-phosphate isomerase	2.76	825799	825905
Spy_1142	-	hypothetical protein	8.28	840838	840914
Spy_1144	-	hypothetical protein	8.33	902140	902304
Spy_1153	-	kup system potassium uptake protein	5.21	278549	278721
Spy_1154	*deaD*	ATP-dependent RNA helicase	18.24	1825059	1825316
Spy_1155	*prfC*	peptide chain release factor 3	6.53	1268883	1269129
Spy_1157	*murF*	UDP-N-acetylmuramoylalanine-D-glutamyl-lysine--D-alanyl-D-alanine ligase	2.75	344517	344698
Spy_1158	*ddl*	D-alanyl-alanine synthetase A	2.94	1265566	1265820
Spy_1167	-	lead, cadmium, zinc and mercury transporting ATPase	4.13	93460	93690
Spy_1169	*spd3*	DNase	4.64	492597	492831
Spy_1209	-	DNA replilcation protein	5.9	954062	954354
Spy_1233	*xseA*	exodeoxyribonuclease VII large subunit	2.31	1236542	1236707
Spy_1264	-	ribose operon repressor	3.86	126617	126863
Spy_1266	-	ATP-dependent protease La	10.25	518270	518346
Spy_1269	*asnA*	asparagine synthetase AsnA	2.73	1385594	1385782

Spy_1280	-	two-component sensor kinase	3.32	260833	261015
Spy_1290	-	hypothetical protein	4.69	151657	151983
Spy_1307	-	hypothetical protein	3.34	849818	849894
Spy_1307	-	hypothetical protein	2.59	551812	551990
Spy_1309	-	sugar transporter permease	2.85	1375304	1375499
Spy_1310	-	sugar transporter permease	3.37	1512396	1512533
Spy_1322	-	hypothetical protein	2.89	1808547	1808709
Spy_1327	*comFA*	competence protein ComF	7.41	1410215	1410373
Spy_1341	*gmk*	guanylate kinase	3.65	643928	644097
Spy_1353	-	hypothetical protein	3.77	274705	274894
Spy_1358	*nadE*	nicotinate phosphoribosyltransferase	2.22	639288	639462
Spy_1363	-	amino acid ABC transporter permease	4.25	53651	53821
Spy_1389	-	sodium-dependent phosphate transporter	5.39	843915	844087
Spy_1390	-	hypothetical protein	4.15	8698	8787
Spy_1403	-	copper chaperone	3.18	418844	419008
Spy_1415	*sdaD2*	phage-encoded DNase	3.28	1137443	1137570
Spy_1434	-	phage protein	2.68	511936	512019
Spy_1439	-	portal protein	4.02	336616	336701
Spy_1439	-	portal protein	2.31	1557961	1558161
Spy_1440	-	terminase large subunit	3.04	1668645	1668851
Spy_1479	*manL*	PTS system mannose-specific transporter subunit IIAB	9.09	177515	177676
Spy_1496	*phaB*	enoyl-CoA hydratase	2.08	649597	649759
Spy_1501	-	N-acetylmuramoyl-L-alanine amidase	3.82	343362	343570
Spy_1503	-	phosphoglycerate mutase	8.01	1285057	1285190
Spy_1504	-	hypothetical protein	2.85	1459480	1459606
Spy_1512	*codY*	transcription repressor CodY	4.8	1267634	1267818
Spy_1513/1514	*-/-*	aminotransferase/universal stress protein	7.14	623952	624056
Spy_1517	-	lantibiotic transport permease	6.59	299394	299543
Spy_1518	-	transporter	2.64	608240	608304
Spy_1519	*recG*	ATP-dependent DNA helicase RecG	4.39	1809352	1809487
Spy_1531	*isp2*	hypothetical protein	5.78	1118092	1118286
Spy_1534	*secA*	preprotein translocase subunit SecA	2.43	1699632	1699788

Spy_1550/1551	*uvrA/corA*	excinuclease ABC subunit A/magnesium and cobalt transporter	3.07	1136156	1136308
Spy_1557	*mutY*	A/G-specific adenine glycosylase	2.22	1223795	1224008
Spy_1571/1572	-/-	c3-degrading proteinase/hypothetical protein	2.42	619074	619164
Spy_1578/1579	-/-	Cro/CI family transcription regulator/transcription regulator	2.17	1404035	1404276
Spy_1587/1589	*udp/crgR*	uridine phosphorylase/GntR family transcription regulator	2.23	1035022	1035171
Spy_1596	*glnA*	glutamine synthetase	5.66	215048	215279
Spy_1599	*pgk*	phosphoglycerate kinase	2.93	1472290	1472422
Spy_1638/1639	*lacA.2/lacR.2*	galactose-6-phosphate isomerase subunit LacA/lactose phosphotransferase system repressor	5.87	436984	437147
Spy_1662	*ulaA*	PTS system ascorbate-specific transporter subunit IIC	4.68	454077	454331
Spy_1677	*yajC*	preprotein translocase subunit YajC	3.96	566490	566708
Spy_1684	*ska*	streptokinase	8.41	1002968	1003109
Spy_1686	*relA*	GTP pyrophosphokinase	2.26	1265964	1266088
Spy_1704	*dppA*	dipeptide-binding protein	15.68	1826850	1826969
Spy_1708	*dppE*	dipeptide transport ATP-binding protein	2.92	90525	90699
Spy_1718	*sic1.01*	inhibitor of complement protein	5.23	1700979	1701145
Spy_1720	*mga*	trans-acting positive regulator	5.18	727744	727901
Spy_1735	*speB*	cysteine protease SpeB	4.58	1493793	1493904
Spy_1737	*rgg*	transcription regulator	2.75	1755059	1755240
Spy_1738	*spd*	phage-associated deoxyribonuclease	2.45	1532865	1533008
Spy_1750	-	hypothetical protein	2.11	73561	73696
Spy_1757/1758	-/-	hypothetical protein/dipeptidase B	3.82	1815244	1815407
Spy_1768	*ahpC*	peroxiredoxin reductase (NAD(P)H)	21.03	213966	214090
Spy_1784/1785	-/-	PTS system, trehalose-specific IIBC component/trehalose operon transcription repressor	3.65	11372	11491
Spy_1785	-	trehalose operon transcription repressor	2.42	1188949	1189072

Spy_1793	*nrdD*	anaerobic ribonucleoside triphosphate reductase	9.14	1408326	1408421
Spy_1794	-	hypothetical protein	4.5	1800671	1800884
Spy_1798	*spxA*	Spx family transcription regulator	4.22	1647749	1647895
Spy_1833	-	transposase	3.67	1547644	1547795
Spy_1834	-	hypothetical protein	2.27	1516157	1516311
Spy_1840/1841	*mnmA/sdhB*	tRNA-specific 2-thiouridylase MnmA/L-serine dehydratase	3.04	1328521	1328665
Spy_1841	*sdhB*	L-serine dehydratase	2.79	609	769
Spy_1843	-	transglycosylase	5.77	1837186	1837408
Spy_1847	-	CDP-diacylglycerol--glycerol-3-phosphate 3-phosphatidyltransferase	2.36	1540231	1540365
Spy_1849	-	zinc protease	6.55	57917	58101
Spy_1851	*hasA*	hyaluronan synthase	3.77	292742	292942
Spy_1856	-	glucose uptake protein	3.37	1711190	1711395
Spy_1857	*guaB*	inosine 5'-monophosphate dehydrogenase	6.82	828775	828903
Spy_1857	*guaB*	inosine 5'-monophosphate dehydrogenase	2.57	1451492	1451574
Spy_1865	*htrA*	protease Do	2.2	279840	279919
Enrichment is determined with respect to the mock control. Greater than 2-fold enrichment is considered significant.					
Genes are considered to be associated with a ChIP-seq peak if the translation start site is within 100 bp upstream to 500 bp downstream of the position of maximal enrichment.					
Genes are listed multiple times if more than one ChIP-Seq peak was present within 100 bp upstream to 500 bp downstream of the transcription start site.					
Multiple genes are listed for a single peak if that peak is within 100 bp upstream to 500 bp downstream of more than one gene.					
*Gene locus numbers refer to MGAS5005 genome.					
