# Supplementary material for: Identification of Group A Streptococcus Genes Directly Regulated by CsrRS and Novel Intermediate Regulators
Source: mBio. 2021 Jul 13;12(4):e01642-21. doi: 10.1128/mBio.01642-21 (PMC8406183; doi:10.1128/mBio.01642-21)
Supplement: TABLE S7 [file mbio.01642-21-st007.pdf]

Table S7. Genes indirectly regulated by CsrR that are regulated by novel transcription regulators					
			NanoString Log2-fold change		RNAseq Log2-fold change
**Gene Locus***	**Gene Name**	**Gene Product**	**0195Ω vs. WT**	**Δ0186 vs. WT**	**Δ*csrR* vs. WT**
Spy_0029	*purD*	phosphoribosylamine-glycine ligase	1.04	0.87	-1.25
Spy_0034	-	transcription regulator	0.49	1.09	1.37
Spy_0113	-	transposase	2.54	0.84	7.17
Spy_0158	*opuABC*	glycine betaine-binding protein/glycine betaine transporter permease	1.24	0.47	-1.53
Spy_0352	-	hypothetical protein	2.97	2.83	6.33
Spy_0356	*speJ*	exotoxin type J	0.23	1.43	1.84
Spy_0551	*rplS*	50S ribosomal protein L19	0.14	-1.29	-1.77
Spy_0652	-	hypothetical protein	0.63	1.38	-1.53
Spy_0858	*xpt*	xanthine phosphoribosyltransferase	0.95	1.51	-1.59
Spy_0978	-	Na(+)-linked D-alanine glycine permease	1.75	1.14	-1.06
Spy_1079	-	PTS system cellobiose-specific transporter subunit IIC	3.15	1.01	1.47
Spy_1170	-	hypothetical protein	-0.06	2.15	1.15
Spy_1238	*artQ*	arginine transporter permease	1.65	1.02	-1.16
Spy_1274	-	acetyltransferase	1.81	0.98	1.28
Spy_1401	-	putative PTS system, enzyme IIA component	4.09	0.93	1.11
Spy_1472	*hit*	bis(5'-nucleosyl)-tetraphosphatase (asymmetrical)	0.18	1.74	1.52
Spy_1528	-	ferrichrome-binding protein	1.13	1.3	-1.83
Spy_1540	*endoS*	endo-beta-N-acetylglucosaminidase F2	2.96	-0.33	-1.29
Spy_1573	*glpF.2*	glycerol uptake facilitator protein	0.09	1.14	-1.06
Spy_1687	*sclA*	hypothetical protein	3.32	0.35	7.75
Spy_1733	-	hypothetical protein	4.12	1.19	3.69
Spy_1839	-	MutT/nudix family protein	1.07	-0.06	-1.15
*Gene locus numbers refer to MGAS5005 genome.					
