# Supplementary material for: Identification of Group A Streptococcus Genes Directly Regulated by CsrRS and Novel Intermediate Regulators
Source: mBio. 2021 Jul 13;12(4):e01642-21. doi: 10.1128/mBio.01642-21 (PMC8406183; doi:10.1128/mBio.01642-21)
Supplement: FIG S1 [file mbio.01642-21-sf001.pdf]

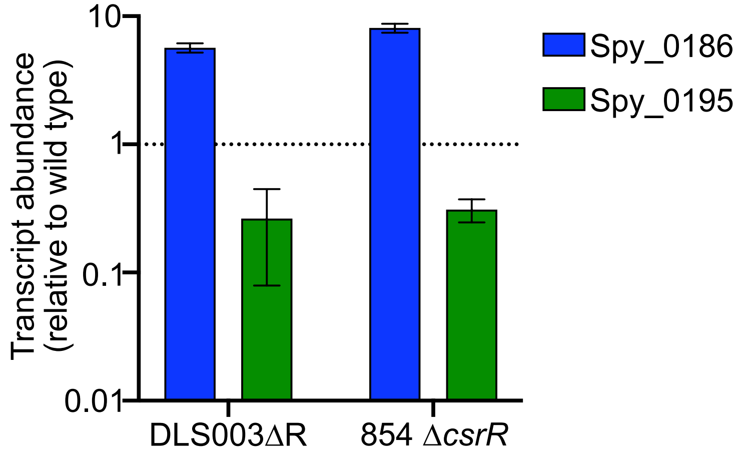

Figure S1. Regulation of *Spy_0186* and *Spy_0195* by CsrR in GAS strains 854 and DLS003. Data represent fold-change in transcript abundance of *Spy_0186* and *Spy_0195* as assessed by qRT-PCR in strain DLS003ΔR relative to that in DLS003 (left) or in strain 854Δ*csrR* relative to that in 854 (right). Values are mean±SEM of duplicate samples of three biological replicates.
