# Supplementary material for: Identification of Group A Streptococcus Genes Directly Regulated by CsrRS and Novel Intermediate Regulators
Source: mBio. 2021 Jul 13;12(4):e01642-21. doi: 10.1128/mBio.01642-21 (PMC8406183; doi:10.1128/mBio.01642-21)
Supplement: TABLE S8 [file mbio.01642-21-st008.pdf]

**Table S8. NanoString analysis of CsrR-regulated genes at mid-exponential phase and the contribution of RopB to regulation**				
**Gene Locus***	**Gene Name**	**Gene Product**	**Log2-fold change Δ*csrR* vs. WT**	**Log2-fold change Δ*ropB* vs. WT**
Spy_0034	-	transcription regulator	1.11	1.77
Spy_0113	-	transposase	7.63	0.14
Spy_0115	-	hypothetical protein	10.21	1.32
Spy_0139	*nga*	NAD glycohydrolase	3.06	1.43
Spy_0140	*ifs*	NADase inhibitor	2.81	1.41
Spy_0141	*slo*	streptolysin O	2.87	1.8
Spy_0142	-	hypothetical protein	2.22	2
Spy_0143	-	hypothetical protein	11.49	-0.22
Spy_0144	-	hypothetical protein	10.52	1.36
Spy_0157	*opuAA*	glycine betaine transport ATP-binding protein	-2.74	1.96
Spy_0158	*opuABC*	glycine betaine-binding protein/glycine betaine transporter permease	-2.12	1.4
Spy_0180	-	murein endopeptidase	1.56	0.67
Spy_0186	-	transcription regulator	2.66	2.09
Spy_0195	-	MarR family transcription regulator	-1.71	1.71
Spy_0275	-	serine/threonine transporter SstT	-1.67	0.88
Spy_0281	-	hypothetical protein	1.02	1.08
Spy_0282	*csrR*	response regulator	-8.69	0.26
Spy_0341	*scpC/prtS*	IL-8 protease	4.58	0.18
Spy_0351	*spyA*	C3 family ADP-ribosyltransferase	3.27	1.33
Spy_0352	-	hypothetical protein	7.29	2.12
Spy_0354	-	hypothetical protein	3.51	0.61
Spy_0355	-	hypothetical protein	6.6	2.13
Spy_0356	*speJ*	exotoxin type J	2.16	0.77
Spy_0357	-	hypothetical protein	2.35	0.8
Spy_0500	-	N-acetylmuramoyl-L-alanine amidase	1.07	1.13
Spy_0551	*rplS*	50S ribosomal protein L19	-1.38	-1.87
Spy_0561	*epf*	extracellular matrix binding protein	3.38	-1.26
Spy_0563	*sagB*	Streptolysin S synthesis protein	0.99	-3.16
Spy_0598	*mscL*	large-conductance mechanosensitive channel	-0.96	-2.49
Spy_0635	*rpmA*	50S ribosomal protein L27	-1.19	-1.95
Spy_0639	*pyrR*	putative pyrimidine regulatory protein	-1.79	-1.32
Spy_0640	*pyrP*	putative uracil permease	-1.56	-0.45
Spy_0651	*bspA*	cell surface protein	-1.01	0.68
Spy_0652	-	hypothetical protein	-1.19	0.5
Spy_0668	*mac*	IgG-degrading protease	7.42	-0.12

Spy_0777	-	hypothetical protein	3.34	0.39
Spy_0858	*xpt*	xanthine phosphoribosyltransferase	-0.98	1.27
Spy_0859	-	xanthine permease	-0.98	1.36
Spy_0996	*speA2*	enterotoxin	2.35	2.52
Spy_1106	*grab*	protein G-related alpha 2M-binding protein	2.79	-0.32
Spy_1114	-	hypothetical protein	1.75	0.21
Spy_1169	*spd3*	DNase	1.07	0.83
Spy_1170	-	hypothetical protein	1.03	0.45
Spy_1237	*artP*	arginine transport ATP-binding protein	-1.16	0.47
Spy_1238	*artQ*	arginine transporter permease	-1.35	0.58
Spy_1274	-	acetyltransferase	1.74	-1.36
Spy_1290	-	hypothetical protein	1.44	-0.44
Spy_1291	-	ATP-dependent RNA helicase	2.61	0.79
Spy_1329	*cysM*	putative O-acetylserine lyase	-1.13	0.72
Spy_1386	-	hypothetical protein	-1.36	-1.41
Spy_1401	-	putative PTS system, enzyme IIA component	2.26	-0.11
Spy_1407	-	esterase	2.64	2.25
Spy_1415	*sdaD2*	phage-encoded DNase	3.54	1.8
Spy_1472	*hit*	bis(5'-nucleosyl)-tetraphosphatase (asymmetrical)	1	0.11
Spy_1473	-	hypothetical protein	1.14	0.2
Spy_1477	-	guanine-hypoxanthine permease	-1.99	0.18
Spy_1531	*isp2*	hypothetical protein	2.12	0.15
Spy_1556	-	hypothetical protein	3.84	0.35
Spy_1557	*mutY*	A/G-specific adenine glycosylase	1.65	0.37
Spy_1680	*pulA*	Pullulanase	0.98	0.09
Spy_1684	*ska*	streptokinase	4.29	1.39
Spy_1704	*dppA*	dipeptide-binding protein	-2.52	0
Spy_1714	-	cell surface protein	1.73	1.32
Spy_1715	*scpA*	C5A peptidase	1.92	1.51
Spy_1718	*sic1.01*	inhibitor of complement protein	3.79	1.88
Spy_1731	-	hypothetical protein	2.45	1.04
Spy_1733	-	hypothetical protein	1.05	-0.84
Spy_1735	*speB*	cysteine protease SpeB	1.18	-2.01
Spy_1738	*spd/speMF*	phage-associated deoxyribonuclease	2.25	-0.34
Spy_1758	-	dipeptidase B	1.81	0.18
Spy_1782	*pepO*	neutral endopeptidase	1.52	0.06
Spy_1851	*hasA*	hyaluronan synthase	7.18	1.41
*Gene locus numbers refer to MGAS5005 genome.				
