# Supplementary material for: Identification of Group A Streptococcus Genes Directly Regulated by CsrRS and Novel Intermediate Regulators
Source: mBio. 2021 Jul 13;12(4):e01642-21. doi: 10.1128/mBio.01642-21 (PMC8406183; doi:10.1128/mBio.01642-21)
Supplement: TABLE S9 [file mbio.01642-21-st009.pdf]

Table S9. NanoString analysis of CsrR-regulated genes at stationary phase and the contribution of RopB to regulation				
**Gene Locus***	**Gene Name**	**Gene Product**	**Log2-fold change Δ*csrR* vs. WT**	**Log2-fold change Δ*ropB* vs. WT**
Spy_0017	-	putative secreted protein	-1.6	1.04
Spy_0034	-	transcription regulator	2.45	-0.08
Spy_0113	-	transposase	3.75	-1.1
Spy_0115	-	hypothetical protein	7.2	-0.88
Spy_0139	*nga*	NAD glycohydrolase	5.03	-0.67
Spy_0140	*ifs*	NADase inhibitor	4.64	-0.58
Spy_0141	*slo*	streptolysin O	4.8	-0.46
Spy_0142	-	hypothetical protein	2.22	-1.56
Spy_0143	-	hypothetical protein	9.19	-1.37
Spy_0144	-	hypothetical protein	8.87	-1.38
Spy_0157	*opuAA*	glycine betaine transport ATP-binding protein	-2.27	-0.04
Spy_0158	*opuABC*	glycine betaine-binding protein/glycine betaine transporter permease	-2.84	-0.49
Spy_0186	-	transcription regulator	2.5	-0.05
Spy_0275	-	serine/threonine transporter SstT	-1.06	0.06
Spy_0281	-	hypothetical protein	2.81	0.81
Spy_0282	*csrR*	response regulator	-7.21	0.81
Spy_0351	*spyA*	C3 family ADP-ribosyltransferase	4.73	-0.03
Spy_0352	-	hypothetical protein	6.11	-0.42
Spy_0355	-	hypothetical protein	1.54	-2.12
Spy_0356	*speJ*	exotoxin type J	1.22	-0.14
Spy_0402	-	hypothetical protein	1.29	-0.43
Spy_0500	-	N-acetylmuramoyl-L-alanine amidase	1.62	0.21
Spy_0501	-	hypothetical protein	1.14	-0.16
Spy_0561	*epf*	extracellular matrix binding protein	3.47	-1.12
Spy_0562	*sagA*	streptolysin S	2.64	-1.06
Spy_0563	*sagB*	Streptolysin S synthesis protein	2.93	-0.55
Spy_0571	-	hypothetical protein	1.93	0.05
Spy_0598	*mscL*	large-conductance mechanosensitive channel	-1.77	-0.64
Spy_0639	*pyrR*	putative pyrimidine regulatory protein	-1.35	0.87
Spy_0640	*pyrP*	putative uracil permease	-1.46	2.07
Spy_0652	-	hypothetical protein	-1.19	-0.12
Spy_0668	*mac*	IgG-degrading protease	3.83	-1.95
Spy_0673	*papS*	tRNA CCA-pyrophosphorylase	1.29	-0.03
Spy_0713	*bcaT*	branched-chain amino acid aminotransferase	-1.14	-0.54
Spy_0777	-	hypothetical protein	2.68	0.21

Spy_0784	*yesN*	putative two-component sensor histitdine kinase	1.02	0.12
Spy_0809	*srtE*	conserved hypothetical protein - lantibiotic	1.1	-0.5
Spy_0913	-	putative integrase/recombinase	-1.03	0.4
Spy_0947	*ciaH*	sensor protein	1.25	-0.69
Spy_0996	*speA2*	enterotoxin	3.95	-0.08
Spy_1139	*nagB*	glucosamine-6-phosphate isomerase	-1.31	-0.93
Spy_1169	*spd3*	DNase	2.5	-0.92
Spy_1170	-	hypothetical protein	2.37	-0.87
Spy_1171	-	conserved hypothetical protein, phage associated	1.11	-1.18
Spy_1284	*ccdA*	putative cytochrome C-type biogenesis protein	1.08	-1.26
Spy_1290	-	hypothetical protein	3.41	-0.81
Spy_1291	-	ATP-dependent RNA helicase	3.57	-0.59
Spy_1329	*cysM*	putative O-acetylserine lyase	-1.17	-0.32
Spy_1407	-	esterase	2.16	0.61
Spy_1415	*sdaD2*	phage-encoded DNase	1.49	-0.84
Spy_1472	*hit*	bis(5'-nucleosyl)-tetraphosphatase (asymmetrical)	0.98	-0.05
Spy_1477	-	guanine-hypoxanthine permease	-1	-0.05
Spy_1479	*manL*	PTS system mannose-specific transporter subunit IIAB	-2.13	-0.35
Spy_1499	*grpE*	putative Hsp-70 cofactor	-2.13	-0.02
Spy_1504	-	hypothetical protein	-1.36	-0.71
Spy_1531	*isp2*	hypothetical protein	1.91	0.29
Spy_1540	*endoS*	endo-beta-N-acetylglucosaminidase F2	1.09	0.75
Spy_1556	-	hypothetical protein	3.75	0.54
Spy_1557	*mutY*	A/G-specific adenine glycosylase	1.28	0.05
Spy_1601	-	membrane protease	1.92	0.59
Spy_1635	*lacD.2*	putative tagatose 1,6-diphosphate aldolase	-2.11	0.07
Spy_1636	*lacC.2*	putative galactose-6-phosphate isomerase (C subunit)	-1.77	0.17
Spy_1637	*lacB.2*	putative galactose-6-phosphate isomerase (B subunit)	-1.38	0.42
Spy_1638	*lacA*	galactose-6-phosphate isomerase subunit LacA	-1.92	0.4
Spy_1684	*ska*	streptokinase	4.65	-0.32
Spy_1687	*sclA*	hypothetical protein	1.27	-2.39
Spy_1702	*smeZ*	mitogenic exotoxin Z	1.26	-0.39
Spy_1704	*dppA*	dipeptide-binding protein	-1.21	-0.1
Spy_1714	-	cell surface protein	2.98	-0.03

Spy_1715	*scpA*	C5A peptidase	3.84	-0.18
Spy_1718	*sic1.01*	inhibitor of complement protein	6.14	-0.04
Spy_1719	*emm1.0*	M protein	1.94	0.63
Spy_1731	-	hypothetical protein	1.6	0.96
Spy_1737	*ropB*	transcription regulator	-1.49	-7.46
Spy_1738	*spd/speMF*	phage-associated deoxyribonuclease	1.7	-0.95
Spy_1825	-	PadR family transcription regulator	-2.75	-0.88
Spy_1851	*hasA*	hyaluronan synthase	3.13	-2.06
Spy_1857	*guaB*	inosine monophosphate dehydrogenase	-1.76	-0.51
*Gene locus numbers refer to MGAS5005 genome.				
